# High-resolution African HLA resource uncovers *HLA-DRB1* expression effects underlying vaccine response

**DOI:** 10.1038/s41591-024-02944-5

**Published:** 2024-05-13

**Authors:** Alexander J. Mentzer, Alexander T. Dilthey, Martin Pollard, Deepti Gurdasani, Emre Karakoc, Tommy Carstensen, Allan Muhwezi, Clare Cutland, Amidou Diarra, Ricardo da Silva Antunes, Sinu Paul, Gaby Smits, Susan Wareing, HwaRan Kim, Cristina Pomilla, Amanda Y. Chong, Debora Y. C. Brandt, Rasmus Nielsen, Samuel Neaves, Nicolas Timpson, Austin Crinklaw, Cecilia S. Lindestam Arlehamn, Anna Rautanen, Dennison Kizito, Tom Parks, Kathryn Auckland, Kate E. Elliott, Tara Mills, Katie Ewer, Nick Edwards, Segun Fatumo, Emily Webb, Sarah Peacock, Katie Jeffery, Fiona R. M. van der Klis, Pontiano Kaleebu, Pandurangan Vijayanand, Bjorn Peters, Alessandro Sette, Nezih Cereb, Sodiomon Sirima, Shabir A. Madhi, Alison M. Elliott, Gil McVean, Adrian V. S. Hill, Manjinder S. Sandhu

**Affiliations:** 1 Centre for Human Genetics, University of Oxford, Oxford, UK; 2 Big Data Institute, Li Ka Shing Centre for Health Information and Discovery, University of Oxford, Oxford, UK; 3 Institute of Medical Microbiology and Hospital Hygiene, University Hospital of Düsseldorf, Heinrich Heine University Düsseldorf, Düsseldorf, Germany; 4 Genome Informatics Section, Computational and Statistical Genomics Branch, National Human Genome Research Institute, Bethesda, MD USA; 5 Wellcome Sanger Institute, Cambridge, UK; 6 Medical Research Council/Uganda Virus Research Institute and London School of Hygiene & Tropical Medicine Uganda Research Unit, Entebbe, Uganda; 7 South African Medical Research Council Vaccines and Infectious Diseases Analytics Research Unit, University of the Witwatersrand, Johannesburg, South Africa; 8 Groupe de Recherche Action en Santé (GRAS) 06 BP 10248, Ouagadougou, Burkina Faso; 9 Center for Vaccine Innovation, La Jolla Institute for Immunology, La Jolla, CA USA; 10 National Institute for Public Health and the Environment, Bilthoven, The Netherlands; 11 Microbiology Department, John Radcliffe Hospital, Oxford University NHS Foundation Trust, Oxford, UK; 12 Histogenetics, New York, USA; 13 Department of Integrative Biology, University of California at Berkeley, California, CA USA; 14 Avon Longitudinal Study of Parents and Children at University of Bristol, Bristol, UK; 15 Population Health Sciences, Bristol Medical School, University of Bristol, Bristol, UK; 16 MRC Integrative Epidemiology Unit, University of Bristol, Bristol, UK; 17 Department of Infectious Disease, Imperial College London, London, UK; 18 The Jenner Institute, University of Oxford, Oxford, UK; 19 The Department of Non-communicable Disease Epidemiology, London School of Hygiene and Tropical Medicine London, London, UK; 20 MRC International Statistics and Epidemiology Group, London School of Hygiene and Tropical Medicine London, London, UK; 21 Tissue Typing Laboratory, Cambridge University Hospitals NHS Foundation Trust, Cambridge, UK; 22 Radcliffe Department of Medicine, University of Oxford, Oxford, UK; 23 Department of Medicine, University of California, San Diego, La Jolla, CA USA; 24 Department of Epidemiology & Biostatistics, School of Public Health, Imperial College London, London, UK

**Keywords:** Genetics research, Vaccines

## Abstract

How human genetic variation contributes to vaccine effectiveness in infants is unclear, and data are limited on these relationships in populations with African ancestries. We undertook genetic analyses of vaccine antibody responses in infants from Uganda (*n* = 1391), Burkina Faso (*n* = 353) and South Africa (*n* = 755), identifying associations between human leukocyte antigen (HLA) and antibody response for five of eight tested antigens spanning pertussis, diphtheria and hepatitis B vaccines. In addition, through HLA typing 1,702 individuals from 11 populations of African ancestry derived predominantly from the 1000 Genomes Project, we constructed an imputation resource, fine-mapping class II HLA-DR and DQ associations explaining up to 10% of antibody response variance in our infant cohorts. We observed differences in the genetic architecture of pertussis antibody response between the cohorts with African ancestries and an independent cohort with European ancestry, but found no in silico evidence of differences in HLA peptide binding affinity or breadth. Using immune cell expression quantitative trait loci datasets derived from African-ancestry samples from the 1000 Genomes Project, we found evidence of differential *HLA-DRB1* expression correlating with inferred protection from pertussis following vaccination. This work suggests that *HLA-DRB1* expression may play a role in vaccine response and should be considered alongside peptide selection to improve vaccine design.

## Abstract (web summary)

Genome-wide analyses of vaccine antibody responses in 2,499 infants from Uganda, South Africa and Burkina Faso identify associations between specific HLA genes and response to eight vaccines, providing insights that could be considered for population-adjusted vaccine design strategies.

## Main

Vaccination is one of the most cost-effective methods for preventing disease caused by infections worldwide^1^. The strategy has been successful in reducing morbidity and mortality associated with multiple infections including diphtheria (a toxin-mediated disease caused by *Corynebacterium diphtheriae*), pertussis (caused by *Bordetella pertussis*) and measles, all of which have vaccines delivered in infancy as part of the Expanded Programme on Immunization (EPI)^2^.

Despite the unquestionable success of vaccination, substantial challenges remain both for maintaining control of vaccine-preventable diseases, and in the development of new vaccines against other diseases. For example, there are increasing reports of epidemics of pertussis in vaccinated communities^3^. These vaccine failures appear to have become more common since the move away from killed whole-cell, to acellular (multi-antigen) pertussis preparations^4^, but the mechanisms underlying the increase in rates of pertussis failures remain unclear, and several countries (particularly in Africa) continue to use whole-cell preparations. Furthermore, it is well recognized that several infectious diseases pose problems for vaccine development including tuberculosis^5^, malaria^6^, human immunodeficiency virus (HIV)^7^ and even severe acute respiratory syndrome coronavirus 2 (SARS-CoV-2), where vaccine breakthrough infections are now widely recognized^8^. Among these diverse challenges, two scientific issues are shared in development pipelines for both established and novel vaccine-preventable diseases. Firstly, the antigens to target and the ideal components of the immune response to stimulate in order to induce protection—so-called correlates of protection—are often difficult to define^9^. Secondly, understanding population differences in risks of vaccine failure is important, particularly in low- and middle-income countries where reporting of failures may not be effectively captured, and where the burden of vaccine-preventable diseases is frequently the highest.

Understanding the impact of human genetic variation has been particularly understudied. It has been recognized for decades that variation across the major histocompatibility complex, known in humans as the *HLA* locus, is associated with differential response and failure to respond to the hepatitis B surface antigen (HBsAg) vaccine^10^, as well as responses against tetanus toxin (TT)^11^ and measles virus (MV)^12^. These findings are in keeping with the well-known association of the locus with susceptibility to multiple other infectious and autoimmune diseases^13–15^. We have recently found that carriage of specific *HLA* gene product alleles (particularly HLA-DQB1*06) is associated with improved SARS-CoV-2 vaccine immunogenicity and may reduce the risk of breakthrough infection with coronavirus disease 2019 after vaccination^16^. Despite detecting these associations, it has not been possible to elucidate the precise causal underlying mechanisms. The presence of *HLA* genes across this locus leads to the speculation that differential peptide binding is responsible. However, the high concentration of genes in the region, the high levels of genetic diversity and epistatic interactions among *HLA* loci within long stretches of linkage disequilibrium (LD) pose substantial challenges to fine-mapping any association signals reliably. Any mapping and downstream mechanistic interpretation is particularly challenging in populations hitherto underrepresented in global genetic studies. Despite statistical and computational advances in HLA biology using methods such as HLA imputation applied to common autoimmune diseases^17,18^, and a limited number of infectious agents such as HIV-1 (ref. ^19^), progress has largely been restricted to populations of European ancestry. Given the worldwide delivery of vaccines, studying vaccine response heterogeneity in African populations offers a series of opportunities. Such work may help to not only understand the influence of host genetics in these understudied populations, but also improve our understanding of vaccine response mechanisms, thus opening avenues for vaccine development for other important infectious diseases.

## Results

### HLA associations with vaccine responses in African infants

**Fig. 1:**
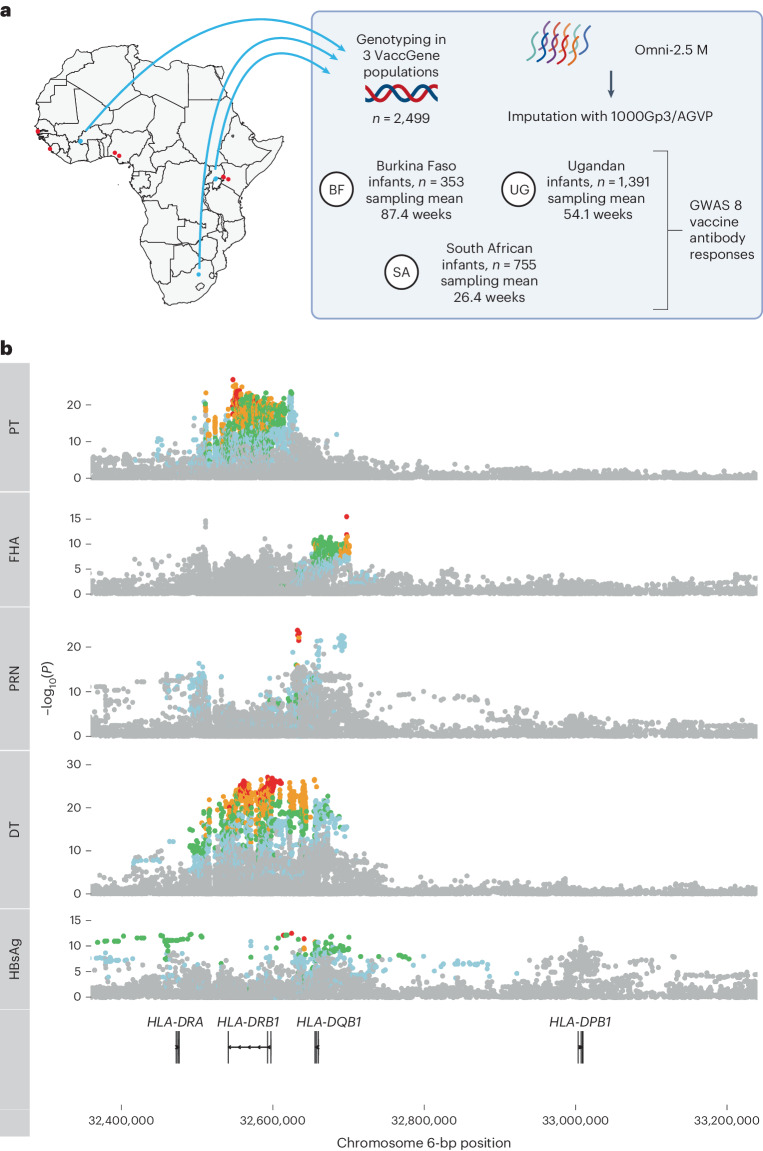
HLA associations with diverse vaccine responses in African infants and the diversity of *HLA* alleles across Africa. **a**, Schematic of the experimental design for the VaccGene project. DNA from 2,499 infants across three African sites were genotyped on the Omni 2.5 M array and then imputed to a merged reference panel of 1000 Genomes phase 3 (1000Gp3) combined with the African Genome Variation Project (AGVP). VaccGene population locations are represented with blue points, and African 1000 Genome populations are in red. The resultant genetic data from VaccGene were tested for association with eight vaccine antibody responses. Created using BioRender.com. The maps of Africa in **a** and Supplementary Fig. 2a were generated using the R packages maps and mapdata (https://CRAN.R-project.org/package=mapdata/) and using data available through the CIA World Data Bank II (2017) release^40^. **b**, Regional association plots of the genetic association statistics from imputed and directly genotyped variants tested for association with five vaccine antigen responses. In each track representing the statistics from a single tested vaccine antibody response, the *y* axis represents the –log_10_(*P* value) of the linear mixed-model genetic association statistics pooled across the three populations, and the *x* axis is the position along chromosome 6 with the units in base-pair coordinates from build 37. The lowest track represents the location of four class II *HLA* genes. No adjustments were made for multiple testing. The bulk of associations can be observed to occur across the *HLA-DRB1* and *HLA-DQB1* regions. Points are colored by LD (*r*^2^) with the index variant in each analysis across all three populations: red (0.8–1), orange (0.6–0.8), green (0.4–0.6), blue (0.2–0.4) and gray (<0.2).

**Extended Data Fig. 1:**
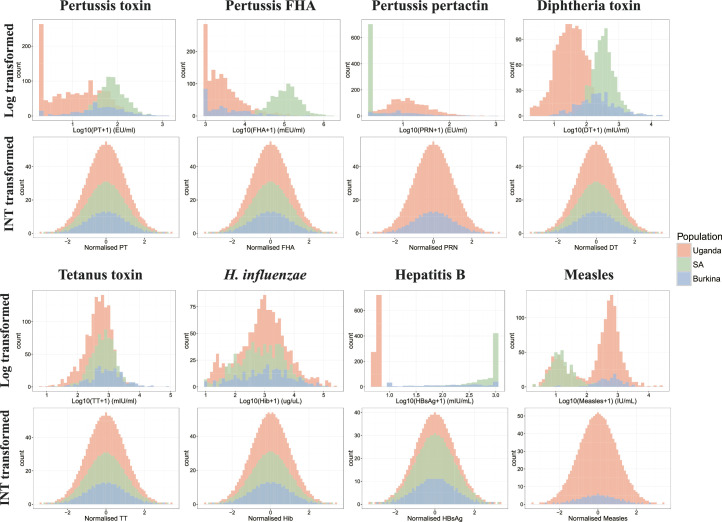
Distributions of vaccine antibody responses in each African population. Distributions of vaccine responses in individual VaccGene cohorts (Uganda n = 1391, South Africa n = 753, Burkina Faso n = 355) following transformation using two methods. Distributions of each vaccine response following either logarithmic (log) or inverse normal transformations (INT; used in the GWAS analyses) are shown for each population separated by colour. Individuals in South Africa (SA) did not receive pertussis pertactin or measles vaccine prior to the antibody assays being performed. Differences in log-transformed distributions are most likely due to differences in timing of sampling as highlighted in Supplementary Table 1.

**Extended Data Fig. 2:**
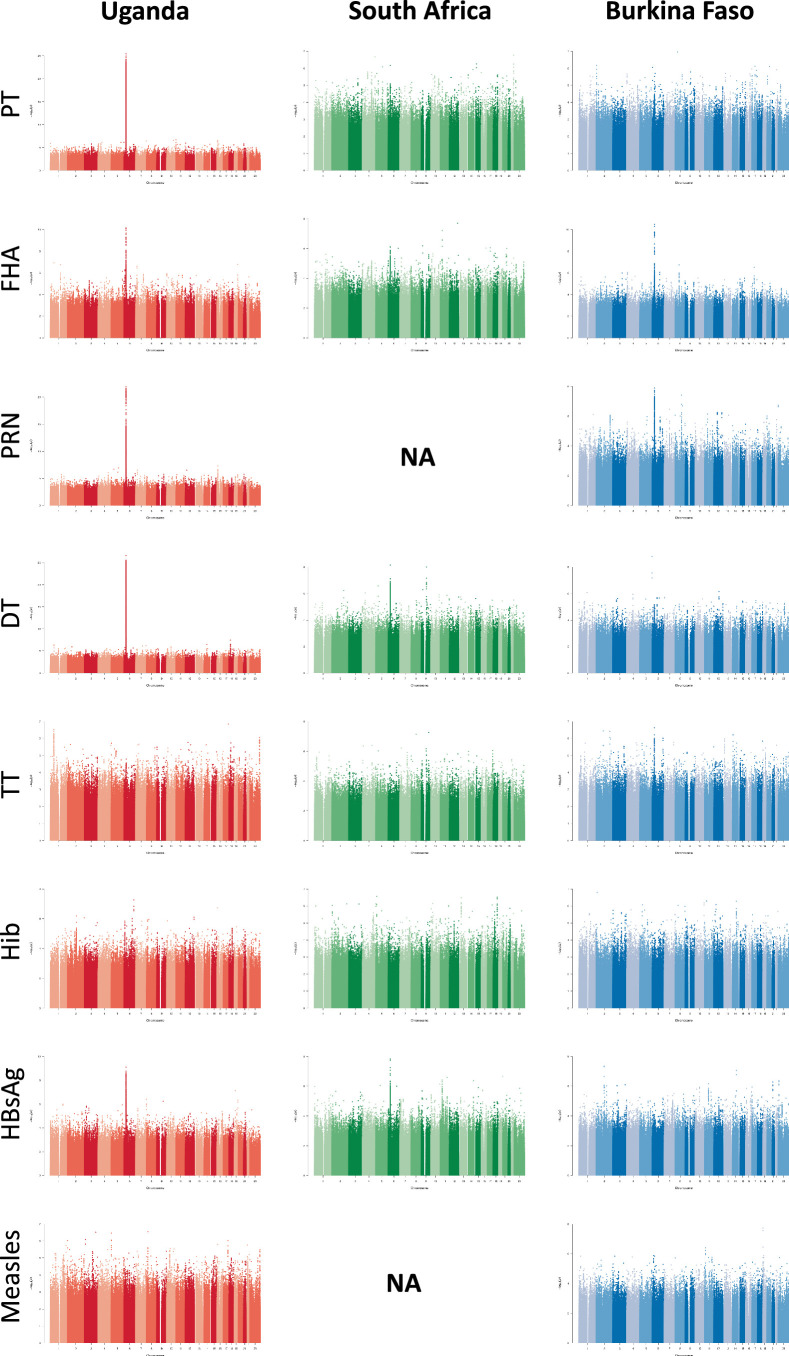
Genetic association plots for each measured antibody and cohort. Manhattan plots of genetic association signals using linear mixed model regression with eight tested vaccine traits in three African populations (Uganda n = 1391, South Africa n = 753, Burkina Faso n = 355). For each plot the x-axis represents the position along the genome from chromosome 1 to 22 and the X chromosome. Each point represents a single variant with the y-axis being –log10(*P*-value).

**Extended Data Fig. 3:**
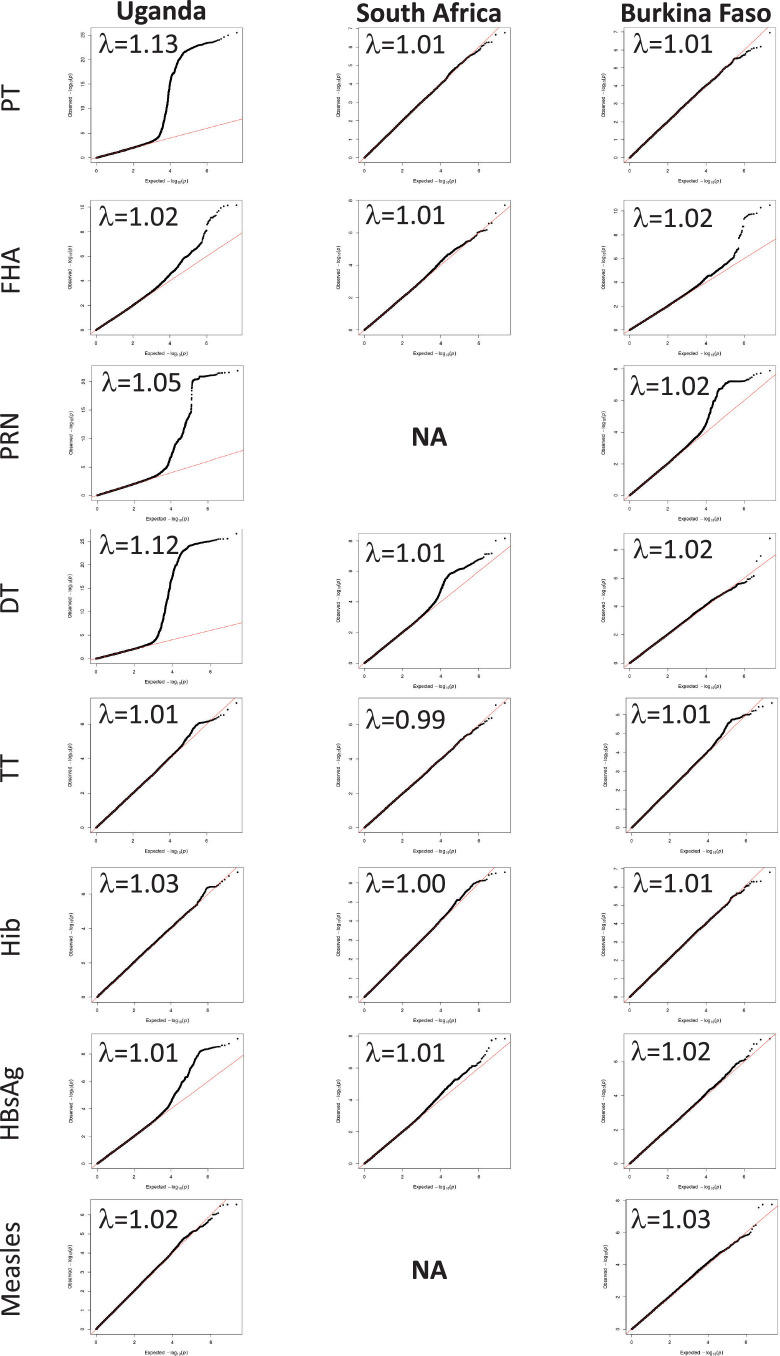
Estimated inflation of association plots excluding the MHC region. QQ plots of the variants tested for association using linear mixed model regression in each individual population and tested antibody response, with the extended MHC region excluded (chromosome 6, 25.5-34 Mb, build 37). The x-axis is the expected *P*-value and the y-axis is the observed with all points representing the tested genetic variants.

We tested for associations between antibody responses against specific vaccine antigens in 2,499 infants recruited from three African countries (Burkina Faso, South Africa and Uganda defined as VaccGene; Fig. 1a). The array and imputation panel^20^ used were designed to allow characterization of genetic variation specific to populations of African ancestries. The vaccine responses measured were immunoglobulin G (IgG) antibody levels against eight vaccine antigens (diphtheria toxin (DT), pertussis toxin (PT), filamentous hemagglutinin (FHA), pertactin (PRN), TT; *Hemophilus influenzae* type b (Hib), MV and HBsAg). The VaccGene population demographics are described in Supplementary Table 1, and a summary of the participating individuals and data quality control (QC) is provided in Supplementary Fig. 1a, Methods and Supplementary Tables 2 and 3. The IgG traits were normalized (using inverse normal transformation; distributions represented in Extended Data Fig. 1). Association testing was performed with time between last vaccine and blood sample included as a fixed-effect covariate, which was shown to be inversely correlated with all traits with response to DT as an exemplar in Supplementary Fig. 1b. A genetic relatedness matrix was included in the association model as a random-effect covariate, and all three cohorts were pooled into a single association model. We identified significant evidence of associations within the HLA region for five vaccine responses including PT, FHA, PRN, DT and HBsAg (Fig. 1b and Supplementary Table 4). All index variants with the smallest *P* value were centered on the class II HLA region and were particularly pronounced across *HLA-DRB1* (for example, rs73727916 for PT, beta = 0.33, *P* = 1.9 × 10^−^^27^) and *HLA-DQ* (rs147857322 for PRN, beta = 0.37, *P* = 4.2 × 10^−^^23^). No associations were observed outside the HLA for any trait, and no associations were observed across the genome for MV or TT responses (Extended Data Figs. 2 and 3).

### High-resolution HLA typing across Africa

We performed high-resolution typing of three class I and eight class II *HLA* genes in 1,702 individuals from African and admixed African American populations. This total included 893 individuals from VaccGene, in addition to 668 individuals from six other African populations (Esan in Nigeria (ESN), Gambian in Western Division, The Gambia – Mandinka (GWD), Luhya in Webuye, Kenya (LWK), Maasai in Kinyawa, Kenya (MKK), Mende in Sierra Leone (MSL), and Yoruba in Ibadan, Nigeria (YRI)), and 141 from two admixed African populations (African Caribbean in Barbados (ACB), and African ancestry in Southwest USA (ASW)) from the 1000 Genomes Project. Newly sequenced individuals from the MKK population were included in this analysis with sample identifiers provided in Supplementary Table 5, and the breakdown of individuals with different data available is provided in Supplementary Table 6.

We compared three different classical HLA typing methods (Sanger sequencing, PacBio long-read sequencing and MiSeq short-read sequencing; Methods) and found that MiSeq offered scalability, accuracy and cost-effectiveness for typing in African populations. Specifically, we found that there was little advantage to using PacBio to detect novel protein-coding variation in *HLA* alleles. Overall, we found that less than 5% of typed individuals in any one population were found to possess novel protein-coding alleles at any locus and this detection was not dependent on typing platform used (Supplementary Fig. 2a and Supplementary Table 7).

**Extended Data Fig. 4:**
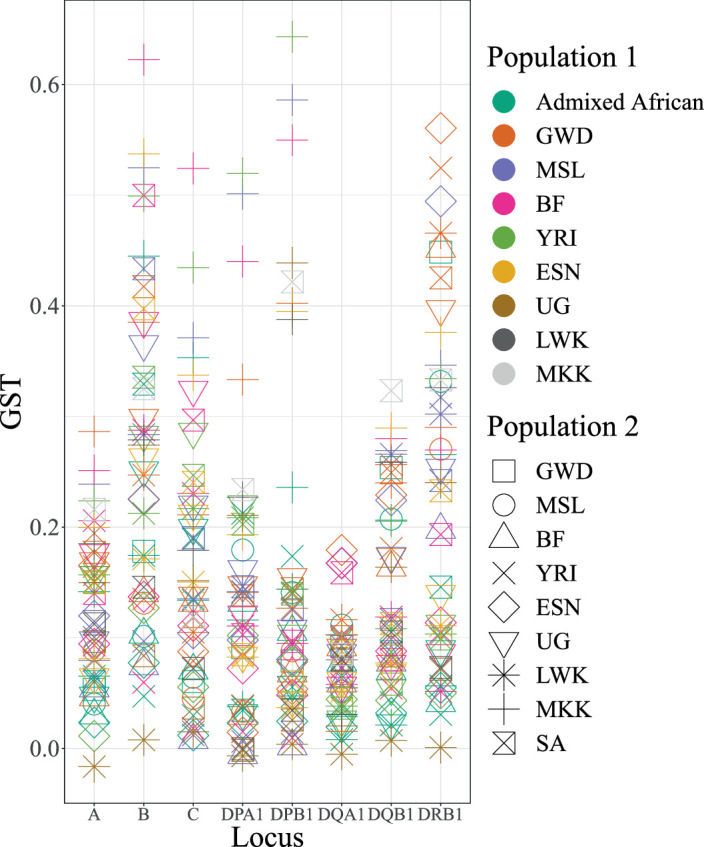
Locus-specific estimates of differentiation between pairs of African populations. Measures of differentiation between African populations for three class I and five class II HLA genes, determined by defining the HLA alleles at 6-digit (3-field) resolution. Estimates, in G_ST_, are between pairs of populations with the first population represented as the colour of each point, and the second as a shape of the point allowing a determination of the combination of populations through colour and shape. Admixed American populations include ACB: African Caribbean in Barbados (n = 79) and ASW: African Ancestry in Southwest USA (n = 62); BF: Burkina Faso (n = 167); ESN: Esan in Nigeria (n = 99); GWD: Gambian in Western Division, The Gambia – Mandinka (n = 112); LWK: Luhya in Webuye, Kenya (n = 97); MKK: Maasai in Kinyawa, Kenya (n = 166); MSL: Mende in Sierra Leone (n = 84); SA: South Africa (n = 396); UG: Uganda (n = 330); YRI: Yoruba in Ibadan, Nigeria (n = 110).

Using pairwise population differentiation estimates, we noted many loci to be substantially differentiated (with a genetic similarilty index (G_ST_ ) greater than 0.4) across the continent including *HLA-B*, *HLA-C* and *HLA-DRB1* (Extended Data Fig. 4). The *HLA-DPB1* locus was particularly differentiated, with some G_ST_ estimates >0.5 equivalent to *HLA-B*, and even observed at the lower two-digit (one-field) level of resolution as shown in the pie charts matched to population geography in Supplementary Fig. 2b. Most *HLA-DPB1* differentiation was observed when comparing against the MKK individuals who also appeared to be differentiated from other tested African populations at *HLA-C* and *HLA-DP* loci (Extended Data Fig. 4). Together, these data support the inclusion of as many different continental populations as possible in any African HLA imputation reference panel.

### A HLA imputation reference panel for Africa

**Extended Data Fig. 5:**
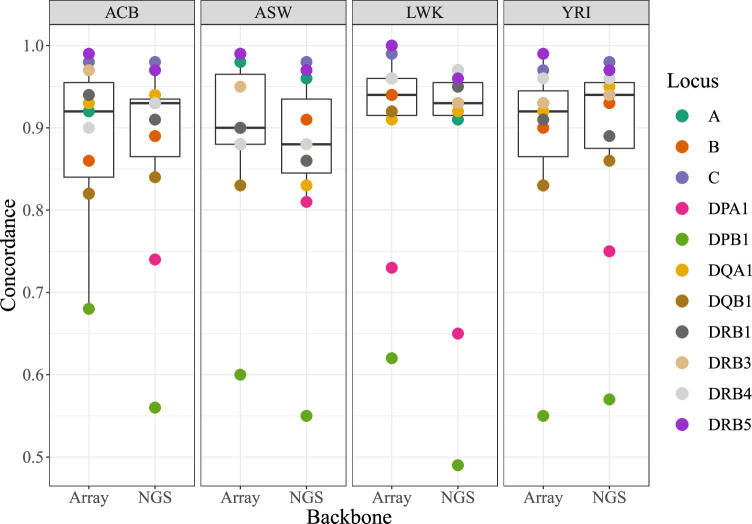
Merging genotyped and sequenced variants across the HLA region for an imputation panel. The first stage in building an imputation panel involved merging variant calls defined through genotyping arrays or next-generation sequencing (NGS). To determine whether imputation calls would differ based on the origin of variant calls we compared imputation performance (using the original HLA*IMP:02 algorithm) in individuals from four African populations with variant data called by array genotyping (Array) or next-generation sequence data (NGS). Points are concordance estimates between imputed and MiSeq called HLA alleles for each gene locus. The box plot centre line represents the median; the box limits, the upper and lower quartiles; and the whiskers are the 1.5x interquartile range. ACB: African Caribbean in Barbados (n = 76); ASW: African Ancestry in Southwest USA (n = 59); LWK: Luhya in Webuye, Kenya (n = 97); YRI: Yoruba in Ibadan, Nigeria (n = 108).

We next combined these high-resolution three-field (six-digit ‘G’) resolution HLA types derived from MiSeq with genotype data from 1,597 individuals across the same 11 African populations to generate a large, comprehensive HLA imputation reference panel available for African populations. We merged variants from both direct array genotyping and next-generation sequencing (NGS) calls including only variants that had a very high (*r*^2^ > 0.999) level of concordance. Using the HLA*IMP:02 algorithm and the original reference panel for imputation^21^, we observed very little difference in allele concordance estimates between *HLA* allele calls derived from either NGS or array genotyping in populations where we had both available (ACB, ASW, LWK and YRI; Extended Data Fig. 5). We did note, however, that concordance estimates were lower for *HLA-DPA1* and *HLA-DPB1* alleles, most likely a result of the poor representation of African DP alleles in the HLA*IMP:02 reference panel.

**Fig. 2:**
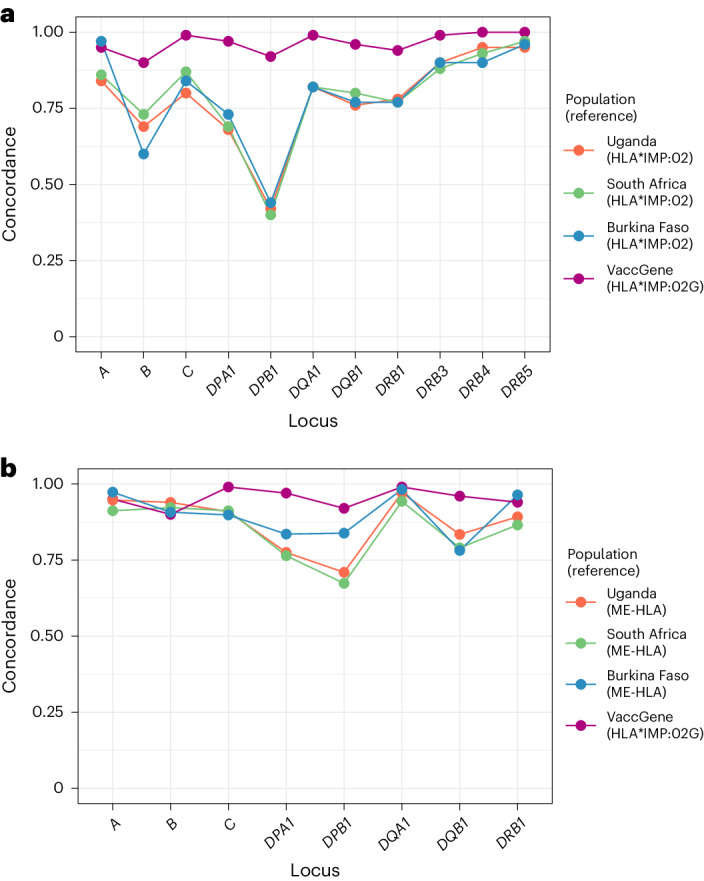
Imputing *HLA* alleles in African populations using a continental reference panel. **a**, HLA imputation performance (measured as locus-specific concordance between alleles called to two-field (four-digit) resolution) in the VaccGene populations using the traditional method and reference set (HLA*IMP:02) clustering by locus and population. Results were compared to the performance of our enhanced high-resolution algorithm and reference dataset (HLA*IMP:02G) using the same individuals divided into validation and test groups using a fivefold cross-validation approach. **b**, HLA imputation performance comparing results from the ME-HLA panel to those from HLA*IMP:02G called to six-digit ‘G’ resolution. Means of performance are plotted as points for each comparison in both plots. Full statistics are available in Supplementary Tables 8–10. A total of 167 individuals with both genotype and HLA data were available from Burkina Faso, 396 from South Africa and 320 from Uganda; 883 individuals were included for the VaccGene comparison.

We proceeded to build the updated imputation panel and algorithm using the merged array/NGS variant calls and incorporating the higher-resolution *HLA* allele calls for African populations, calling this updated system HLA*IMP:02G. We compared three imputation algorithms against MiSeq typing used as the gold standard employing a fivefold cross-validation approach. The algorithms and reference panels were HLA*IMP:02G (MiSeq-derived HLA calls with merged array/NGS variant calls), the original HLA*IMP:02 algorithm with the original multi-ancestry reference panel, and a recently developed multi-ancestry imputation reference panel (the Broad multiethnic HLA panel, ME-HLA)^22^. Only calls to two-field (four-digit) resolution were available for HLA*IMP:02. Overall, we observed a significant improvement in calling of *HLA* alleles at all loci with the updated HLA*IMP:02G algorithm compared to HLA*IMP:02 (Fig. 2a; performance statistics are available in Supplementary Tables 8 and 9). The greatest increase in imputation performance was observed at the *HLA-DPB1* locus, where the mean concordance increased from 0.42 using HLA*IMP:02, to 0.92 using HLA*IMP:02G.

HLA*IMP:02G also outperformed the ME-HLA panel at *HLA-DPB1* and *HLA-DQB1*, but other alleles (*HLA-A*, *HLA-B* and *HLA-DRB1*) were called as effectively using the ME-HLA panel as they were for HLA-IMP:02 G (Fig. 2b; statistics are available in Supplementary Table 10). These results not only support the inclusion of diverse populations in African ancestry-specific reference panels to substantially improve the performance of population-specific *HLA* allele imputation, but also highlight the benefit of targeted typing in some individuals to further refine population-specific signals.

### Fine-mapping HLA associations with vaccine antigen responses

**Fig. 3:**
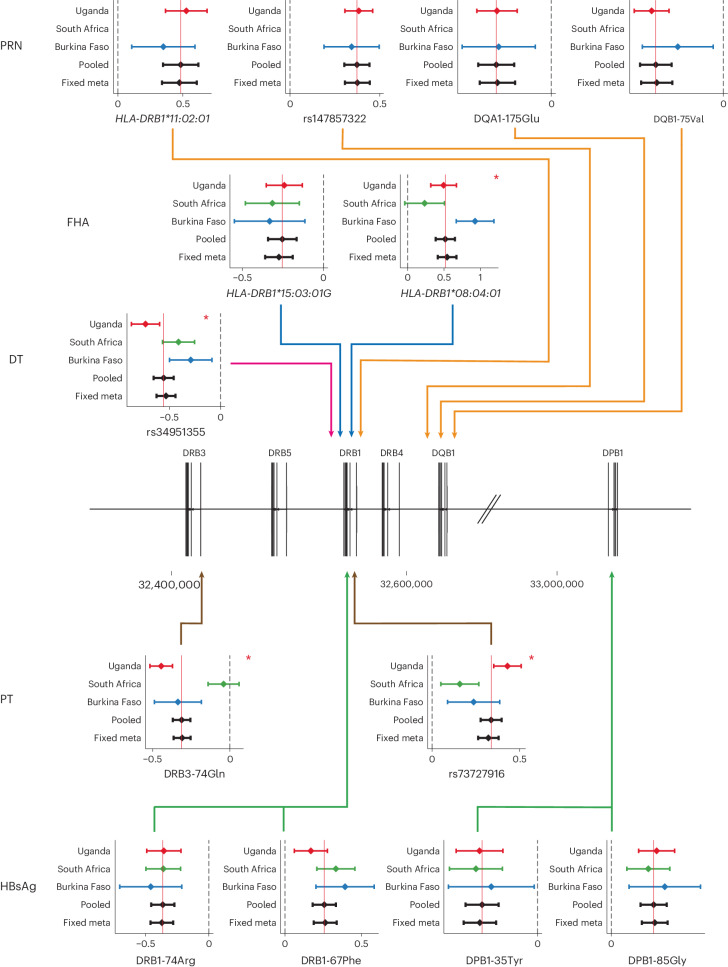
HLA associations with vaccine responses fine-mapped to HLA variants. Forest plots of beta effect estimates from linear mixed-model association tests (center points) for fine-mapped variants for each trait colored by population (Uganda, red; South Africa, green; Burkina Faso, blue) with 95% confidence intervals (bars), followed by corresponding distributions for the pooled linear mixed-model (‘pooled’, solid black horizontal line) and fixed-effects meta-analyses including all cohorts (‘fixed meta’). Variants were identified to be independently associated with each trait using combined manual and automated regression approaches. Dashed vertical black lines represent no effect (beta = 0), and solid vertical red lines cross the beta estimate of the ‘pooled’ model as a reference. The originating locus of association is represented by solid arrowed lines colored by trait indicating the relevant region of association on chromosome 6. Associations demonstrating significant evidence (*P*_Q_ ≤ 1 × 10^−^^3^) of heterogeneity using Cochran’s *Q* test are highlighted with a red asterisk. No adjustments were made for multiple testing. Exact *P* values for the association tests are provided in Supplementary Table 12. PRN was not administered to South African infants; hence, there are no measured effects for this population. Data from 1,320 Ugandan individuals, 716 individuals from South Africa and 309 individuals from Burkina Faso, restricted by relatedness were included in the analysis.

**Extended Data Fig. 6:**
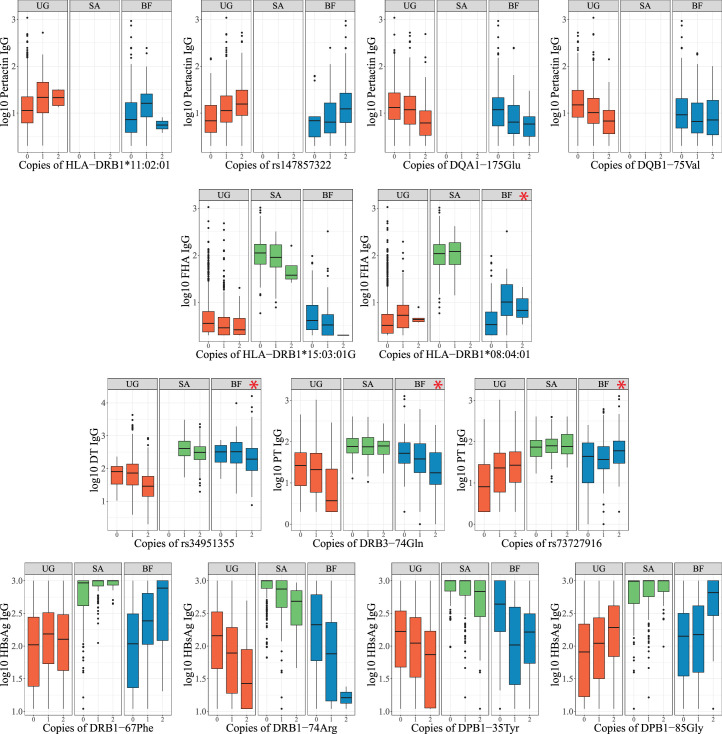
Distributions of antibody responses for 13 genetic associations with vaccine response stratified by variant dosage and population. Distributions of log10 transformed antibody levels against five vaccine antigens are shown for 2345 individuals from Africa stratified by population and dosage of genetic variant detected as most significant using the combined manual and automated regression approach described in the main text, Methods and Fig. 3. The box plot centre line represents the median; the box limits, the upper and lower quartiles; and the whiskers are the 1.5x interquartile range. Associations with significant evidence (*P*_Q_ ≤ 1×10-3) of heterogeneity tested for using the Cochran’s Q test are highlighted with a red asterisk (*), with exact *P*-values provided in Supplementary Table 12. PT: pertussis toxin; FHA: pertussis filamentous hemagglutinin; DT: diphtheria toxin; HBsAg: hepatitis B surface antigen.

We used *HLA* alleles imputed with HLA*IMP:02G to test for association between 71,297 single-nucleotide variants (SNVs), 164 *HLA* alleles and 2,809 HLA amino acid residues with a minor allele frequency > 0.01. We then used a stepwise fine-mapping approach to identify 13 statistically significant (*P*_pooled_ ≤ 5 × 10^−^^9^) associations with each of the vaccine traits mapping to multiple HLA class II loci. Stepwise conditional regression results are shown in Supplementary Figs. 3a–c, and the final results after a combination of manual and automated regression modeling are provided in Fig. 3, with individual statistics provided in Supplementary Tables 11 and 12. Raw allele dosages and phenotype distributions are available in Supplementary Table 13 and Extended Data Fig. 6. We observed that each of the traits exhibited multiple, independent association signals that were best explained by *HLA* alleles, SNVs or amino acids each in different *HLA* genes. For DT, for example, we found that the same SNV as identified in the first round of analysis (rs34951355) provided the smallest *P* value and explained the association most parsimoniously. In contrast, PT was best explained by two independent associations: the same SNV as identified in the genotype-only GWAS (rs73727916, beta_univariate_ = 0.34, *P*_univariate_ = 8.1 × 10^−^^30^), and the presence of the amino acid glutamine at position 74 of *HLA-DRB3* (DRB3-74Gln, beta_univariate_ = −0.32, *P*_univariate_ = 2.0 × 10^−^^29^). For some associations, the difference in association statistics between alleles in linkage was small (particularly those occurring in *HLA-DRB1* and the DRB pseudogenes *DRB3*, *DRB4* and *DRB5*), and so evidence for true causal association remains circumstantial.

**Fig. 4:**
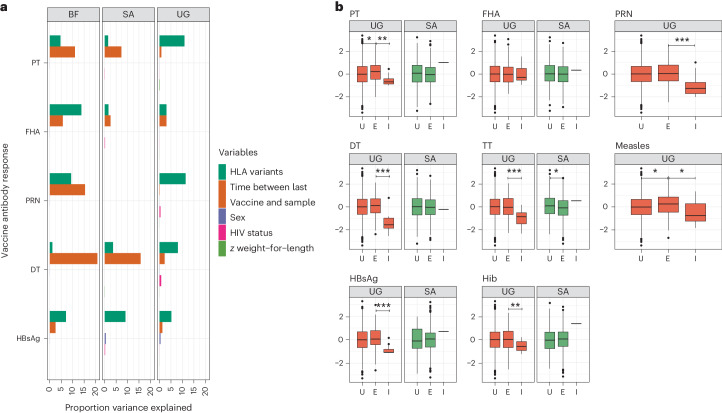
Assessing the impact of genetics and other exposures on magnitude of vaccine response in VaccGene. **a**, The proportion of variance explained (*r*^2^) by genetic variants (those fine-mapped to be most relevant as in Fig. 3 for each antibody trait), time in weeks between last vaccine and sampling for antibody assay, sex (male versus female), HIV status and weight-for-length *z*-score at birth, were available in each tested cohort. Data from 1,391 Ugandan (UG), 755 South African (SA) and 355 Burkinabe (BF) individuals were included in the analysis. **b**, Distributions of antibody responses stratified by HIV status (uninfected (U), exposed (E) or infected (I) at birth) at birth in Ugandan and South African individuals with differences tested between strata using the two-tailed Wilcoxon rank test. The exact *P* values for differences between ‘E’ and ‘I’ groups in Uganda for each vaccine are 1.0 × 10^−^^4^ (PT), 0.7 (FHA), 1.3 × 10^−^^5^ (PRN), 8.4 × 10^−^^8^ (DT), 8.5 × 10^−^^5^ (TT), 8.1 × 10^−^^3^ (Hib), 0.01 (Measles) and 1.2 × 10^−^^4^ (HBsAg). Numbers of individuals per group and log-transformed data distributions are available in Supplementary Table 14. No adjustment for multiple testing was applied to any of the reported statistical associations. In the box plots, the center line represents the median, the box limits denote the upper and lower quartiles, and the whiskers are 1.5 times the interquartile range. *P < 0.05, **P < 0.01, ***P < 0.001.

**Extended Data Fig. 7:**
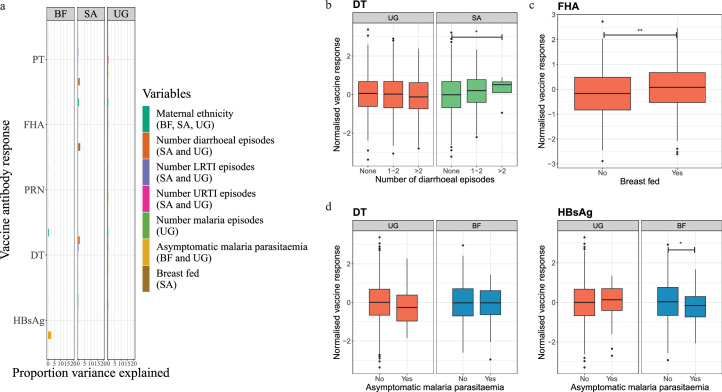
Assessment of other exposures on magnitude of vaccine response in VaccGene. (**a**) The proportion of variance explained (*r*^2^) by variables including self-reported maternal ethnicity (including only groups containing 20 or more individuals), number of diarrhoeal episodes reported between birth and blood sampling for vaccine response measurement, number of lower respiratory tract infections, number of upper respiratory tract infections, number of episodes of malaria, presence of asymptomatic parasitaemia at the point of blood sampling and whether or not the infant was breast fed before sampling. The cohorts in which the variables were available are listed in the legend. (**b**) Distributions of antibody responses against DT stratified by number of diarrhoeal episodes between birth and sampling in Ugandan (UG) and South African (SA) individuals with test of significance calculated using linear regression, *P* in SA 0.02. (**c**) Distributions of antibody responses against FHA stratified by breast feeding status before blood sampling in UG individuals with test of significance performed using linear regression, *P* = 0.01. (**d**) Distributions of antibody responses against DT and HBsAg stratified by presence of asymptomatic parasitaemia at point of sampling for vaccine response in Ugandan (UG) and Burkinabe (BF) individuals with test of significance undertaken using linear regression. The *P*-value in BF individuals testing for HBsAg is 0.03. The box plot centre line represents the median; the box limits, the upper and lower quartiles; and the whiskers are the 1.5x interquartile range. All plots include data from 1391 Ugandan, 755 South African and 355 Burkinabe individuals. Differences in (b) to (d) tested using a 2-tailed Wilcoxon rank test. No adjustment for multiple testing was applied to any of the reported statistical associations. * *P* < 0.05; ** *P* < 0.01.

We next used other data available from the infants recruited in Uganda, Burkina Faso and South Africa to understand the proportion of variance of antibody responses explained by diverse variables compared to host genetics. Variables available in all cohorts included time between vaccination and sampling (included as a covariate in all genome-wide association studies (GWAS) models), sex, weight-for-length *z*-score at birth and HIV status. We found that the contribution of genetic variation consistently outweighed the impact of other measured variables except for time between vaccination and sampling (Fig. 4a). Overall, we observed little effect of sex and weight-for-length variables on antibody variance, when measured at the point of sampling for antibody measurements, or of HIV status at birth, although we did observe that the small number of individuals infected with HIV at birth in Uganda had lower levels of antibody against most vaccine responses (Fig. 4b; distributions of antibody are in Supplementary Table 14). We tested a range of other variables suspected to contribute significantly to variable antibody responses but found they explained less than 2% of the variance in each of the tested cohorts (Extended Data Fig. 7). The mean proportion of variance explained by the HLA variants across the three tested populations was 5.7% (range 1.5–10.9%) for PT, 6.1% (1.6–13.8%) for FHA, 10.4% (9.3–11.4%) for PRN, 4.3% (1.2–7.0%) for DT and 7.1% (5.2–9.1%) for HBsAg.

### Correlating immunogenicity and effectiveness through HLA

**Fig. 5:**
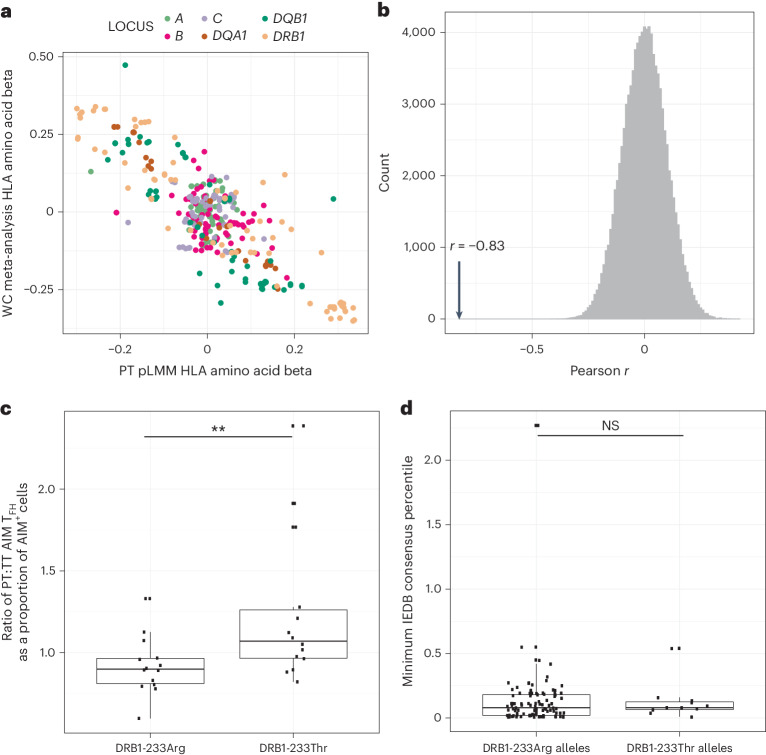
Mechanisms associated with HLA-mediated responses and vaccine failure. **a**, The beta effect estimates for association between HLA amino acid residues and PT antibody response in the 2,499 VaccGene infants plotted against the equivalent estimates from a meta-analysis of two case–control association studies including 5,066 individuals with records of self-reported pertussis that leads to whooping cough. WC, whooping cough. **b**, Distributions of Pearson’s *r* coefficient following 100,000 permutations to measure the significance of correlation between effect estimates of HLA amino acids pruned by LD comparing responses against PT and against the pertussis GWAS. Pearson correlation coefficients were calculated after relabeling of the pertussis GWAS variants generating the null distribution. The correlation coefficients determined using the true datasets are represented with a vertical arrow. **c**, Ratio of circulating PT:TT-specific T_FH_ cells in donors of known *HLA-DRB1* type divided into *HLA-DRB1* variant associated with PT antibody response and pertussis self-report, DRB1-233Arg *n* = 15, and DRB1-233Thr *n* = 14. Antigen-specific T_FH_ cells are represented as a proportion of all cells categorized as antigen inducible marker (AIM^+^) cells. A one-tailed Mann–Whitney test was used to test for a statistical difference between groups; *P* = 0.007. **d**, Predicted affinities for top PT-derived peptides predicted to bind to alleles with those containing a threonine at position 233 of *HLA-DRB1* (‘DRB1-233Thr’, *n* = 11) compared to those with an arginine (‘DRB1-233Arg’, *n* = 101) calculated from the Immune Epitope Database (IEDB), with a difference tested using a two-tailed Mann–Whitney test. In the box plot, the center line represents the median, the box limits denote the upper and lower quartiles, and the whiskers are 1.5 times interquartile range. **P < 0.01; NS, not significant.

**Extended Data Fig. 8:**
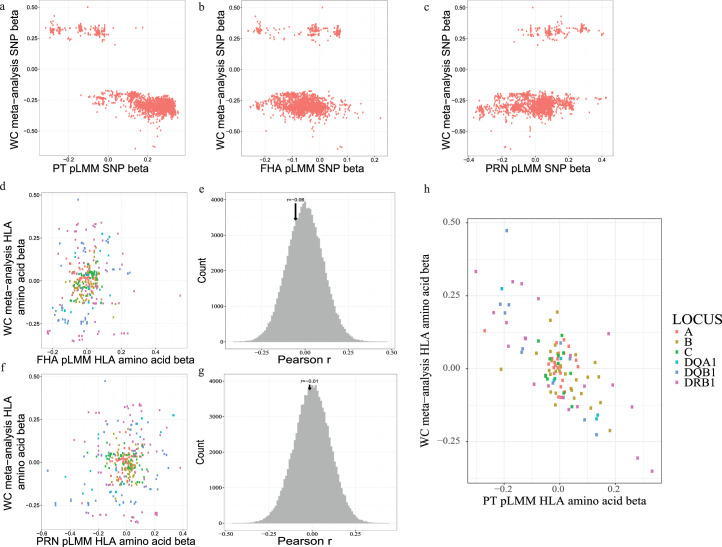
Correlating signals of HLA association with pertussis vaccine response and infection. (**a** - **c**) Correlation of SNV beta effect estimates derived from GWAS of self-reported pertussis (causing whooping cough) and GWAS of antibody responses measured in African infants against PT (a), FHA (b) and PRN (c). Estimates were not available for pertussis GWAS for SNPs with *P* > 1×10-5. Two-tailed Pearson’s r coefficients of correlation were determined to be -0.86 (a), 0.19 (b) and 0.38 (c). (**d**-**g**) Correlation of HLA amino acid residue beta effect estimates derived from GWAS of self-reported whooping cough and GWAS of antibody responses against FHA (d) and PRN (f). Residues are coloured by HLA gene. The distributions of measured Pearson *r* following 100,000 permutations to measure the significance of correlation between effect estimates of HLA amino acids pruned by LD (*r*2 < 0.35) comparing responses against FHA (e) and PRN (g) are also shown. (**h**) The beta effect estimates for association between HLA amino acid residues and PT antibody response in the VaccGene infants are plotted against the equivalent estimates from a whooping cough GWAS following pruning of the residues by LD (r2 < 0.35). Residues are coloured by HLA gene. WC, whooping cough.

Given the observed impact of genetic variants on antibody response, we next aimed to understand these genetic associations in the context of vaccine effectiveness. A large independent case–control genetic association study of self-reported pertussis (a disease with a characteristic whooping cough) is available and was undertaken using data from vaccinated adolescents and young adults in the United Kingdom who had received pertussis vaccine^23^. We compared the results from our genetic association analysis investigating pertussis antibody responses against the results from the available pertussis GWAS. We observed a statistically significant negative correlation between the effect estimates for SNVs (Extended Data Fig. 8a) and amino acid residues (Fig. 5a) only when using the results from our PT responses. For amino acid residues, Pearson’s *r* was estimated at −0.83 (*P*_perm _< 1 × 10^−8^ after 108 permutations; Fig. 5b). No correlation was observed for the two other pertussis antigens (Extended Data Figs. 8b–g). The observed amino acid correlation with PT persisted after stringent correction for LD (Extended Data Fig. 8h).

These data provide evidence that the genetic architectures of PT responses and self-reported pertussis are negatively correlated, altogether suggesting that PT may be a correlate of efficacy against pertussis.

### HLA allelic impact on T_FH_ cell activity

We next tested whether the observed PT association exerted effects through the antigen presentation–T cell immunological axis. To achieve this, we first had to identify the most likely causal, index variant affecting both PT response and pertussis susceptibility. The results from our fine-mapping in Africa revealed an *HLA-DRB3* variant as being most significant, whereas an *HLA-DRB1* variant was highlighted as most associated with self-reported pertussis in the UK populations. We performed dedicated imputation of HLA-DRB1 and DRB3 in the Avon Longitudinal Study of Parents and Children (ALSPAC) cohort and observed a negative correlation in effect estimates between ALSPAC self-reported pertussis and the pooled PT effect estimates for HLA-DRB1 amino acids determined from the infants recruited in Uganda, Burkina Faso and South Africa (*r* = −0.55, *P*_perm_ < 1 × 10^−^^5^) but little evidence of correlation for HLA-DRB3 (*r* = 0.13, *P*_perm_ = 0.16). Together, these results suggest that the functional variant is most likely to reside in *HLA-DRB1*. The most significantly associated *HLA-DRB1* variant in both studies is amino acid position 233, in linkage with both the HLA-DRB3 amino acid residue (*r*^2^ = 0.50 in African populations) and rs73727916 (*r*^2^ = 0.54). Amino acid position 233 may be an arginine (DRB1-233Arg) or a threonine (DRB1-233Thr) associating with lower or higher PT antibody responses in the Ugandan cohorts, respectively.

We used this DRB1-233 residue to stratify individuals from independent studies into two groups and compared levels of antigen-specific follicular helper T (T_FH_) cells (Supplementary Fig. 4 and Supplementary Table 15)^24^. We found that individuals carrying DRB1-233Thr had, on average, a 1.2-fold greater ratio of PT:TT-specific T_FH_ cells compared to individuals carrying DRB1-233Arg (one-tailed Mann–Whitney *P* = 0.007; Fig. 5c). Despite these associations, we found no evidence of differences in the affinity (Fig. 5d) or breadth (Supplementary Table 16) of PT peptide binding defined by DRB1-233 using in silico peptide binding methods.

### *HLA* expression loci correlate with vaccine responses

**Extended Data Fig. 9:**
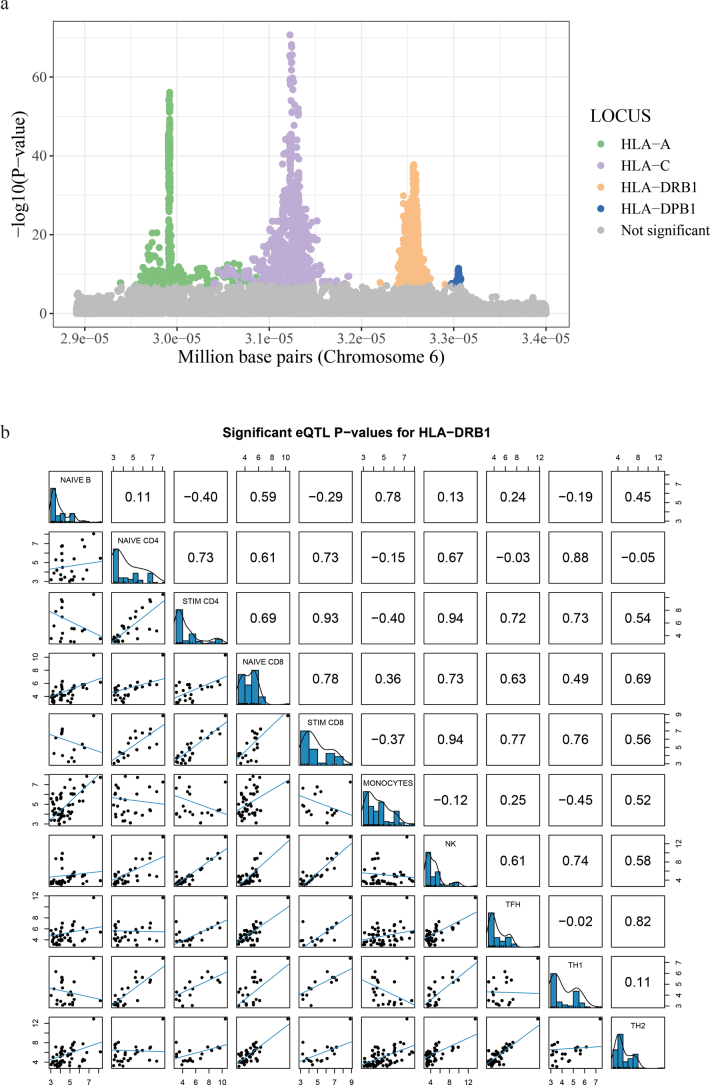
Correlating HLA gene cis-eQTL effects between peripheral immune cell types. (**a**) Variants with evidence of being cis-expression quantitative trait modulators are plotted by position across the HLA against evidence of significance of impacting expression of four HLA transcripts. Only variants with significant evidence (*P* < 5 ×10^-8^) of association from the meta-analysis of estimates derived from linear regression performed in each population group are coloured by gene, with the remainder of variants coloured in grey. RNA sequence data from lymphoblastoid cell lines from 655 individuals were mapped to personalised HLA gene sequences derived from high-resolution typing. (**b**) The correlation in *P*-value estimates for variants predicted to be cis-eQTL variants in different cell types from 80 individuals included in the DICE dataset. 10 of 13 cell types are presented with scatter plots in the lower half of the table and two-tailed Spearman *rho* estimates in the upper half. Included cells are naïve B-cells, naïve and stimulated (STIM) CD4, and CD8 T-cells, monocytes, natural killer (NK), and follicular helper (T_FH_), helper-1, and helper-2 T-cells.

Because we had found evidence that HLA binding may not be the predominant mechanism driving an activation of antigen-specific T cells, we next aimed to test whether *HLA* gene expression may play a role in driving these traits. We developed two expression quantitative trait loci (eQTL) resources to test this hypothesis. Firstly, we combined HLA-wide SNV genotypes with RNA-sequencing (RNA-seq) data derived from immortalized lymphoblastoid cell lines (LCLs) from many of the same individuals included from our imputation reference panel (655 from six African populations recruited as part of 1000Gp3, and *cis*-expression summary statistics provided in Extended Data Fig. 9a and Supplementary Table 17). The second resource focused on the cell-specific impact of variants using a published ex vivo cell-specific eQTL dataset including 13 cell types from 80 individuals (Extended Data Fig. 9b)^25^.

**Fig. 6:**
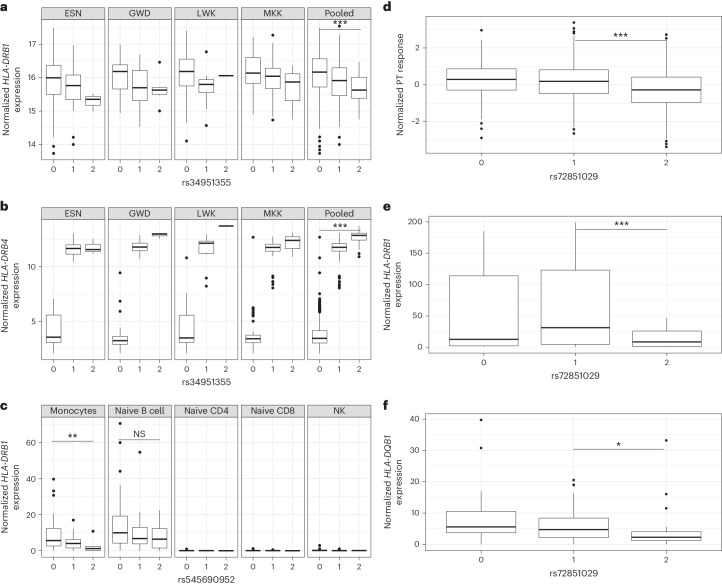
Mapping *cis*-eQTL across the HLA in diverse immune cells. **a**, Effect of the index variant (rs34951355) associated with differential DT response in African infants on normalized *HLA-DRB1* expression in immortalized LCLs from individuals from four African populations (99 individuals from ESN, 112 from GWD, 97 from LWK and 166 from MKK). Only those with more than a single observation in each genotype category are shown. A plot of the data from the pooled set of four populations is also shown. The *x*-axes numbers refer to the number of copies of the C allele compared to the A allele in each group of individuals per population. Differences between allele copies within the final pooled set were calculated using unadjusted linear regression, *P* = 4.7 × 10^−^^5^. **b**, The effect of the same variant on normalized *HLA-DRB4* expression in the same individuals as detailed in **a**, *P* = 1.6 × 10^−^^215^. **c**, The effect of rs545690952 (a variant in LD with rs34951355) on *HLA-DRB1* expression in circulating monocytes, naive B cells, naive CD4^+^ and CD8^+^ T cells and natural killer (NK) cells from 80 individuals with expression and *HLA* allele data available from the DICE study demonstrating a consistent direction of reduced *HLA-DRB1* expression in monocytes with significance tested using linear regression. Monocytes, *P* = 8.6 × 10^−3^. **d**, The effect of alternate T alleles of rs72851029 on PT antibody response in the African infant GWAS, *n* = 2,499 with significance tested in a recessive model using linear regression, *P* = 6.9 × 10^−29^. **e**, The effect of rs72851029 on *HLA-DRB1* expression in monocytes from 80 individuals from DICE with significance tested using a recessive model using linear regression, *P* = 0.001. **f**, The effect of rs72851029 on *HLA-DQB1* expression in monocytes from the same 80 individuals with significance tested between groups using an additive model with linear regression and testing only the difference between individuals carrying one or two T alleles of the variant, *P* = 0.05. In the box plots, the center line represents the median, the box limits denote the upper and lower quartiles, and the whiskers are 1.5 times the interquartile range. ^*^P ≤ 0.05, ***P ≤ 0.001.

**Extended Data Fig. 10:**
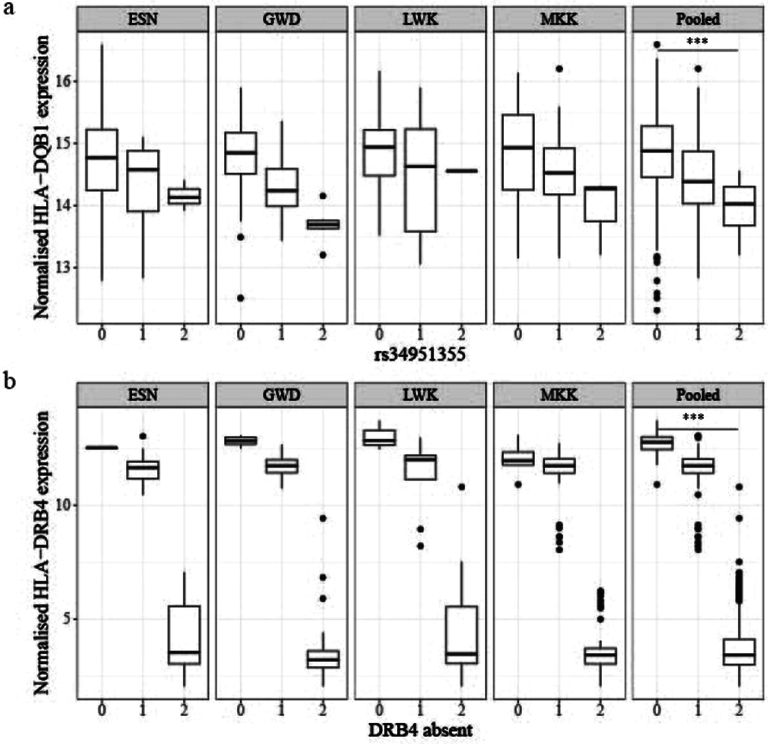
Effects of variants on HLA gene expression. (**a**) Effect of the index variant (rs34951355) associated with differential DT response in African infants on normalised *HLA-DQB1* expression in immortalised lymphoblastoid cell lines from individuals from four African populations (99 individuals from ESN, 112 from GWD, 97 from LWK, and 166 from MKK). Only those populations with more than a single observation in each of the three genotype categories are shown. A plot of the data from the pooled set of four populations is also shown. The x-axes numbers refer to the number of copies of the C allele compared to the A allele in each population. The significance of association in the Pooled set was tested using linear regression, *P* = 5.2×10^-15^. (**b**) The distribution of measured HLA-DRB4 expression in the same number of lymphoblastoid cells lines given the number of carried haplotypes where *HLA-DRB4* is predicted to be absent (that is traditionally not carrying HLA-DRB1*04, *07 or *09 alleles), with significance in the pooled set tested using linear regression, *P* = 1.7×10^-211^. The box plot centre line represents the median; the box limits, the upper and lower quartiles; and the whiskers are the 1.5x interquartile range. * P < 0.05, ** P < 0.01, *** P < 0.001, NS: not significant.

We found that the adenine allele of the DT-associated rs34951355 was associated with downregulated expression of *HLA-DRB1* (*P*_meta_ = 7.1 × 10^−6^; Fig. 6a) and *HLA-DQB1* (*P*_meta_ = 5.2 × 10^−^^15^; Extended Data Fig. 10a) in the immortalized LCLs. The most striking association for this variant, however, was increased expression of *HLA-DRB4* (*P*_meta_ = 1.6 × 10^−215^; Fig. 6b). rs34951355 was the index variant explaining the differential *HLA-DRB4* expression in the lymphoblastoid cell set, indicative of this variant tagging the *HLA-DRB4* haplotype (Extended Data Fig. 10b). In the cell-specific datasets, rs34951355 was not available and thus rs545690952 (*r*^2^ of 0.80 with rs34951355 in the African datasets, *P*_pooled_ = 3.0 × 10^−27^ with DT response) was used instead and found to be associated with both differential *HLA-DRB1* (*P* = 6.3 × 10^−3^; Fig. 6c) and *HLA-DQB1* expression in monocytes in the same negative direction, consistent with a cell-specific effect in one of the most critical antigen-presenting cells in the circulation. *HLA-DRB4* gene expression data were not available for this cell-specific dataset. Although we observed variant-specific differences in *HLA-DRB1* and *HLA-DRB4* expression, we again could not identify an antigen-specific difference in the in silico predicted breadth of peptide binding for DRB4-linked *HLA-DRB1* alleles, or other alleles not found on DRB4 haplotypes (Supplementary Table 18).

In light of these findings for DT, and the inconsistent results for PT peptide binding, we next tested whether the PT association may also be related to differential *HLA* gene expression. In the cluster of variants associated with PT, rs72851029 was most significantly associated with decreased PT antibody response (*P*_pooled_ = 6.6 × 10^−25^; Fig. 6d), decreased *HLA-DRB1* expression in the LCLs from African individuals (*P*_meta_ = 1.25 × 10^−22^), decreased *HLA-DRB1* (*P* = 5.0 × 10^−4^; Fig. 6e) and *HLA-DQB1* (*P* = 0.05; Fig. 6f) expression in monocytes. Furthermore, in an independent analysis of cell surface protein expression on monocytes from Sardinian individuals, this same rs72851029 variant was also associated with reduced expression of HLA-DR (beta effect on the thymine allele −0.61, *P* = 8.1 × 10^−46^)^26^.

To test whether eQTL variation may be, at least in part, responsible for the observed variation in PT responses, we used Bayesian information criterion (BIC) manual regression to compare alternative models, similar to the approach used for fine-mapping outlined in Fig. 3. In such modeling, a lower BIC value is deemed to suggest a better fit to the model. Together, the rs73727916 and DRB3-74Gln variants (Fig. 3) yielded a BIC of 5,527.2. An alternative model including only rs72851029 did not fit the model so well (BIC of 5,551.0), but when including the index eQTL variant for *HLA-DRB1* (derived through the immortalized lymphoblastoid cells), rs9270645, and the DRB1-233Thr amino acid variant, a better fit to the model was achieved (BIC of 5,519.9). Altogether, these data provide further evidence that *HLA-DRB1* expression alongside some other allele-specific effect may play a major role in influencing pertussis and diphtheria antibody responses, as well as potentially in the risk of pertussis following vaccination with acellular pertussis vaccine.

## Discussion

In this work, we found that HLA variation is significantly associated with antibody responses against five vaccine antigens delivered to African infants. Using an HLA imputation resource with high-resolution MiSeq typing, we fine-mapped the signals of association to numerous class II HLA variants and alleles. We found *HLA-DRB1* variants to be associated with increased PT-specific T_FH_ cell activity, increased antibody production and ultimately protection against pertussis. However, we found less evidence of an effect mediated through predicted binding but instead more evidence of an effect mediated through *HLA* gene expression, which we also observed for DT responses.

Together, our results highlight the importance of human genetic variation influencing responses against multiple vaccines delivered to infants worldwide that until now have only been appreciated reproducibly for vaccinations targeting SARS-CoV-2 (ref. ^16^, hepatitis B virus (HBV)^27,28^, meningitis C^11^, measles^29^ and HIV^30^. Class II HLA associations are particularly well characterized for HBV and SARS-CoV-2 vaccine responses, consistent with similar associations observed for susceptibility or outcomes following infection with many diseases including HIV^19^ and tuberculosis^31^. We used classical HLA typing in a large proportion of our study individuals to improve confidence in downstream imputation in African populations. We observed improvements in imputation performance through including individuals from multiple diverse populations in our HLA*IMP:02G algorithm and reference panel, although it is important to note that the ME-HLA imputation resource that has recently been released^22^, performed equivalently at multiple loci of anticipated medical importance. We found *DPA1* and *DPB1* loci to be particularly differentiated across the continent, which could have relevance for traits such as HBsAg response, given our observed HLA-DP associations consistent with previous reports^27,32,33^, and other viral infections including SARS-CoV-2 (ref. ^34^) and HIV-1 (ref. ^30^).

The high-resolution HLA calls also facilitated high-confidence eQTL calls for *HLA* genes. Differential expression of *HLA-C* has been linked with susceptibility to HIV disease progression^35^ but, to our knowledge, there have been no previous reports linking class II *HLA* expression and infection-related traits. Furthermore, despite increasing availability of datasets for characterizing the impact of variants on HLA gene and protein expression, few are specific to Africa. Our HLA eQTL resources highlight the potential importance of *HLA* expression on vaccine responses possibly acting in isolation, as we postulate for DT acting through *HLA-DRB1* and *HLA-DRB4* expression, or alternatively through a combination of expression and peptide binding effects as observed for PT as increasingly recognized in autoimmunity^36^. Although compelling, these results highlight the importance of future method development to colocalize *HLA* expression and peptide binding datasets, accounting for the complex structure of the HLA region, to understand the functional and clinical implications of these effects.

For those vaccine responses where we found a genetic association, we observed that genetics explained up to 10% of the variance of antibody responses, second only to timing between vaccination and blood sampling. Strikingly, we did not observe a significant difference for any vaccine response when stratified by sex. This observation contrasts with reports from other studies investigating diphtheria^37^ and HBV^38^, where antibody responses have been noted to be higher in young females. The reasons for these inconstancies are unclear, but as the timing of sampling for the historical diphtheria observation was 8 weeks after vaccination, whereas ours was 7 or 8 months after their last vaccine dose, we hypothesize that sex effects may have been observed if we sampled closer to the time of vaccination.

The clinical relevance of our work is multifold. Firstly, in light of our observed *HLA* expression effects, and given some vaccine adjuvant effects may in part be due to increased *HLA* expression^39^, alternate adjuvant selection based on expression boosting for infections such as pertussis could help achieve more universal protection. Secondly, given the recognized *HLA* allele frequency variation by population, it is likely that these HLA associations could have greater relevance for some populations over others. Risks of breakthrough infection may be more common in some populations owing to genetic differences; thus, consideration of these differences may be important for future vaccine delivery. Finally, if human genetic variation impacts the effectiveness of other vaccines that we are striving to develop, such as HIV for example, then it is even more important to identify such associations a priori before making statements about population-scale vaccine effectiveness.

Our work does have several limitations. The heterogeneous nature of the tested cohorts could affect the interpretation of our results. We observed significant heterogeneity for some association signals including the index HLA-DR signal observed with PT, where a null association was observed for the South Africa cohort, which remains unexplained. It may be due to the use of acellular rather than whole-cell vaccine in South Africa, which is the only obvious difference in vaccine delivery, or could be as a result of a yet-unidentified genetic, environmental or other population difference. Our results also highlight the ongoing challenges with reliably fine-mapping HLA association signals across such diverse populations. Exemplified for pertussis, the most likely causal variant defined by the fine-mapping in our VaccGene cohort was a HLA-DRB3 amino acid residue, but, when combining our data with those of a related phenotype from a UK dataset, we found near-equivalent evidence that the signal was instead linked to a *HLA-DRB1* variant that could equally alter peptide binding or gene expression. These are recognized challenges with the *HLA* locus, and we propose that we will only be able to overcome these challenges through improved resource availability, increased power and use of multiple approaches to reliably pinpoint the underlying mechanisms. It is also important to note the self-reported nature of the pertussis phenotype used for correlation with the antibody measures^23^. Self-report will be less specific than depending on a clinical test or diagnosis and will be subject to recall bias. Nevertheless, the striking correlation observed for PT, rather than PRN or FHA suggests that self-report is likely to be a reasonable marker of the memorable whooping cough typical of pertussis. Finally, we used IgG antibody for our measured trait for association, but there are many other potential readouts that could be used for the vaccines tested. IgA subtypes may have been more appropriate for *Hib* and microneutralization for measles, and the use of IgG alone may explain the absence of association for these tested vaccine responses.

In conclusion, our results demonstrate that variation of *HLA* gene expression is likely to play a role as part of a multifaceted set of mechanisms influencing important biological processes. Resources such as our collective African genetic and transcriptomic datasets may be key to understanding multiple genetic associations across the HLA with traits of importance across Africa within a functional context.

## Methods

### Experimental design and study populations

The objectives of this study were to (1) test for association between genetic variation and antibody response to eight vaccine antigens delivered in infancy, (2) characterize the major *HLA* genes in a large collection of African populations using a range of sequence technologies, (3) use this resource to develop and test a population-specific HLA imputation panel, and (4) use the high-resolution characterization to understand the likely functional mechanisms underlying these measured vaccine responses. The African populations included in this study include seven populations characterized as part of 1000Gp3, the Maasai from the HapMap collection and three other populations recruited as part of the VaccGene initiative with separate details of ethical approvals provided below. The analyses used genotype data, described in more detail below, derived from array-based and/or NGS data alongside *HLA* allele information for all included populations. Association analyses were undertaken using only VaccGene populations incorporating array-derived genotype data alongside *HLA* allele types, vaccine antibody responses and clinical demographic data.

#### Statistics and reproducibility

We estimated that a sample size of 2,500 individuals would have 94.7% power to identify variants explaining 2% of the variance of antibody responses with a *P*-value threshold of observing an association due to chance (*α*) of 1 × 10^−8^ using the Genetic Power Calculator (http://zzz.bwh.harvard.edu/gpc/). We explicitly aimed to measure and analyze quantitative traits that did not require randomization or blinding for generating the data or comparing through analyses. Data from samples with prespecified poor data quality were excluded as detailed in the relevant sections below. No other criteria for exclusion were applied to any other experiment. In any experiment where group assignment may have been possible (for example, the flow experiments comparing T_FH_ frequencies between carriers of different HLA-DRB1 amino acid residues), flow and bioinformatic analysts were blinded to sample status until the final plotting and comparison stage.

#### 1000Gp3 and HapMap collections

The collection, genotyping and sequencing of the seven 1000Gp3 African populations have already been described and all data are publicly available including more recently available high-coverage, whole-genome sequencing data (http://www.internationalgenome.org/). These data include individuals from ACB, ASW, ESN, GWD, LWK, MSL and YRI populations. DNA was extracted from samples of publicly available immortalized LCLs selected from unrelated individuals from these 1000Gp3 populations and from the MKK population derived from the HapMap project^41^. The resultant DNA was used for short-read and long-read HLA gene sequencing and typing. DNA from the MKK population was also sequenced across the genome using short-read sequencing with all methods described below.

#### VaccGene populations

Participants included in the VaccGene study were recruited from three African countries selected partly due to their geographic dispersal across the continent and partly due to the availability of high-quality metadata and biological samples relevant to infant vaccination. These sites were in Uganda, South Africa and Burkina Faso. Individuals from each of the cohorts were included if their dates of birth, vaccination and blood sampling were available and if it was confirmed that they had received three doses of vaccines including DT, TT, pertussis antigens, Hib and HBsAg, and a single dose of MV vaccine. The receipt of vaccines was confirmed through referencing the vaccination cards of infant participants or the documented administration of vaccines by the research teams where relevant. Beyond exclusion criteria involved in preliminary recruitment of the individuals, no further exclusion occurred based on gender, ethnicity, HIV exposure or any other health status. A range of clinical and demographic metadata were collected from the three cohorts, including the number of illnesses during the first year of life, details regarding the pregnancy and parental occupations and self-reported ethnicities (Supplementary Table 1). A description of each of these populations is detailed below.

##### Uganda: the Entebbe Mother and Baby Study (EMaBS)

The Entebbe Mother and Baby Study (EMaBS) is a prospective birth cohort that was originally designed as a randomized controlled trial (ISRCTN32849447) to test whether anthelminthic treatment during pregnancy and early infancy was associated with differential response to vaccination or incidence of infections such as pneumonia, diarrhea or malaria (http://emabs.lshtm.ac.uk/)^42^. EMaBS originally recruited 2,507 women between 2003 and 2006; 2,345 live births were documented, and 2,115 children were still enrolled at 1 year of age. Pregnant women in the second or third trimester were enrolled at Entebbe Hospital antenatal clinic if they were resident in the study area, planning to deliver in the hospital, willing to know their HIV status and willing to take part in the study. They were excluded if they had evidence of possible helminth-induced pathology (severe anemia, clinically apparent liver disease, bloody diarrhea), if the pregnancy was abnormal, or if they had already enrolled during a previous pregnancy. The mothers and infants underwent intensive surveillance during the first year of infant life. The primary results of the clinical trial demonstrated that anthelminthic treatment during pregnancy had no effect on infant response to Bacillus Calmette–Guérin, tetanus or measles immunization, or the risk of subsequent infectious diseases^42^. All infants under follow-up had a sample of whole blood collected annually on or around their birthday (2–5 ml depending on the age)^43^. The child’s samples were subsequently divided into plasma and red cell pellets as detailed below. Infants were included in the present study if (1) receipt of three doses of DTwP/Hib/HBV (at approximately 6, 10 and 14 weeks of age) and one dose of MV vaccine (at 9 months of age) could be confirmed as being administered by the research team or from their vaccination records, (2) DNA could be extracted from stored red cell pellets, and (3) plasma samples were available from the 12-month age point of sampling. Informed written consent was reacquired from the mothers or guardians, and where appropriate consent from the child and assent from the guardian or mother, specifically for the genetic component of this study. Ethical approval was provided locally by the Uganda Virus Research Institute (ref. GC/127/12/07/32) and Uganda National Council for Science and Technology (MV625), and in the United Kingdom by London School of Hygiene and Tropical Medicine (A340) and Oxford Tropical Research (39-12 and 42-14) ethics committees.

##### South Africa: the Soweto Vaccine Response Study

Six-month-old infants born in Chris Hani Baragwanath Hospital living in the Soweto region of Johannesburg, South Africa were identified from screening logs and databases of participants involved in vaccine clinical trials^44^ coordinated by the Vaccine and Infectious Diseases Analytics (Wits-VIDA) Unit (https://wits-vida.org/). Mothers had originally participated in a randomized, double-blind, placebo-controlled clinical trial in 2011 and 2012 on the safety, immunogenicity and efficacy of trivalent inactivated influenza vaccine during pregnancy, where the trials had demonstrated that the vaccine was immunogenic and provided partial protection against influenza^45^. Mothers of the infants were approached if the infants had received all of their vaccines up to six months of age (DTaP/Hib/HBV at approximately 4, 8 and 12 weeks of age). After receiving information about the study, the mothers were consented in accordance with ethical approval from the University of Witwatersrand Human Research Ethics Committee (ref. M130714) and the Oxford Tropical Research Ethics Committee (1042-13 and 42-14). The infants were sampled prospectively at 6 months of age and at 12 months after receipt of MV vaccine at 9 months. Single whole-blood samples were collected and prepared using a similar protocol to that used in EMaBS to extract DNA from cell pellets and plasma for antibody assays.

##### Burkina Faso: The VAC050 ME-TRAP Malaria Vaccine Trial

Infants between the ages of 6 and 18 months living in the Banfora region of Burkina Faso were recruited into a phase 1/2b clinical trial (NCT01635647) to test the safety, immunogenicity and efficacy of an experimental heterologous viral-vectored prime-boost liver-stage malaria vaccine^46^. These infants were all expected to receive their EPI vaccines (DTwP/Hib/HBV) as part of the usual national schedule at 4, 8 and 12 weeks of age. Infants were precluded from participating in the trial if they were found to have clinical or hematological (venous hemoglobin less than 8 g dl^−1^) evidence of severe anemia, history of allergic or neurological disease or malnutrition. The primary endpoint of the trial has been published demonstrating that the vaccine is safe and immunogenic but had no protective efficacy against clinical malaria^47^. Of 730 infants who were recruited into the study following informed and written consent from the mother, samples suitable for extraction of DNA were collected and stored from 400 infants (350 vaccine recipients and 50 recipients of a control rabies vaccine). Samples of plasma were available from the infants at multiple time points following the experimental vaccine receipt. Samples from individuals taken at time points as close to the 12-month age as possible were prioritized for EPI vaccine response measurements. The infants underwent intensive clinical history and examination during screening and follow-up. The mothers of the participating infants provided consent for their children to be enrolled in the clinical trial and for subsequent genetic studies to be undertaken for all vaccines received in accordance with ethical approval from the Ministere de la Recherche Scientifique et de l’Innovation in Burkina Faso (ref. 2014-12-151) and the Oxford Tropical Research Ethics Committee (41-12).

#### ALSPAC

Genotype data are available from ALSPAC as described previously^23,48,49^ and selected using the fully searchable data dictionary and variable search tool (http://www.bristol.ac.uk/alspac/researchers/our-data/). Consent for biological samples was collected in accordance with the Human Tissue Act (2004), and ethical approval for the study was obtained from the ALSPAC Ethics and Law Committee and the Local Research Ethics Committees.

### Laboratory methods

#### 1000Gp3 and HapMap DNA extraction

Commercially available plates of DNA extracted from LCLs (ACB: MGP00016; ASW: MGP00015; ESN: MGP00023; GWD: MGP00019; LWK: MGP00008; MSL: MGP00021; YRI: MGP00013) and individual aliquots of DNA from cell lines of MKK samples (Supplementary Table 5) were all acquired from Coriell Institute for Medical Research.

#### VaccGene blood sampling and preparation

Whole blood was sampled into vacutainer tubes (BD, Becton Dickinson and Company) containing ethylenediaminetetraacetic acid (for the Ugandan and South African studies) or lithium heparin (Burkinabe) as an anticoagulant. Following centrifugation, the samples were separated into their constituent parts (plasma, buffy coat and erythrocyte layers) and stored at −80 °C until downstream analysis in batches. DNA was extracted from the erythrocyte layer in the Ugandan study and from the buffy coat in South African and Burkinabe studies. DNA from all cohorts was extracted from the relevant samples using Qiagen QIAamp DNA Mini or Midi Kits (Qiagen) using recommended protocols. Whole blood was also sampled into serum separator tubes (SST; BD) in the Ugandan study and serum was isolated and stored according to the recommended protocols.

#### HLA classical allele typing

##### HLA allele nomenclature and methods used for typing

Throughout this paper, *HLA* alleles were classified according to the World Health Organization Nomenclature Committee for Factors of the HLA System. All alleles have a ‘HLA’ prefix followed by a hyphenated gene name and a subsequent star separator. Two- or three-digit fields of between two to three digits in length each then follow this prefix separated by colons.

Traditionally, HLA calls have been defined based on variation within exons of the genes that encode the peptide binding domains (exons 2 and 3 for class I and exon 2 for class II). Therefore, true sequence diversity across all other exons and introns for each gene is relatively unknown, although reference databases are continually accruing extended sequences for many described alleles. As a consequence of this observation, a ‘six-digit G’ level of resolution has been determined whereby alleles can be suffixed by a ‘G’ donating that the sequence of the exons encoding the peptide binding domain of that gene would be consistent with a ‘group’ of alleles. These groups of alleles are defined according to a list maintained by the IMGT/HLA working group (http://hla.alleles.org/wmda/hla_nom_g.txt; accessed on 20 April 2016).

Although there are substantial data available for worldwide HLA types, many such datasets have been generated using various methodologies spanning sequence-specific oligonucleotide and primer technologies through to Sanger and NGS methods that target variable regions of each gene. A single best allele call is often presented for each chromosome and each individual that often represents a long list of potential ambiguities, and such technologies do not always offer the opportunity to elucidate these ambiguities with challenges in terms of IMGT data releases. With the aid of the increased coverage of exon sequencing possible with the MiSeq platform used in this work and described in more detail below, it was possible to reduce both the lists of potential alleles included in ‘groups’ and reduce *cis*/*trans* ambiguities through phasing with MiSeq sequencing technology. An amended ‘G’ list was therefore developed to account for these differences. In the majority of tested individuals, it was possible to resolve alleles to a single six-digit (three-field) call, whereas in some cases a G code was still required. The exons sequenced for all genes using the MiSeq platform included: exon 2 alone (*HLA-DPA1*, *HLA-DQA1*, *HLA-DRB3*, *HLA-DRB4* and *HLA-DRB5*); exons 2 and 3 (*HLA-DPB1*, *HLA-DQB1* and *HLA-DRB1*); exons 1, 2, 3 and 4 (*HLA-A* and *HLA-B*); and exons 1, 2, 3, 4 and 7 (*HLA-C*).

For a subset of individuals and loci it was possible to undertake near whole-gene PacBio sequencing. Only DNA passing stringent quality and yield (greater than 2 micrograms) thresholds was used for PacBio sequencing, and genes were targeted sequentially resulting in sequential attrition of sample availability biased to specific loci. Genes were prioritized in the following order: *HLA-B*, *HLA-A*, *HLA-C*, *HLA-DQB1*, *HLA-DRB1*, *HLA-DQB1*, *HLA-DPB1*, *HLA-DQA1* and *HLA-DPA1*.

The six-digit ‘G’ resolution HLA typing was performed for all African samples using a commercial platform developed by Histogenetics (Ossining). Whole-gene long-read sequencing was performed using PacBio technology for a subset of African individuals and loci. Exon targeted MiSeq (Illumina) sequencing was performed by Histogenetics (Ossining) following preparation of libraries from individual DNA according to MiSeq protocols with two amplification rounds tagging adaptor and index sequences followed by sequencing on a MiSeq machine according to manufacturer protocols. The resultant fastq files were processed and typed using proprietary HistoS and HistoTyper softwares (Histogenetics)^50^ using IMGT/HLA Release 3.25.0 July 2016. Gene-targeted PacBio sequencing was undertaken by Histogenetics on the RS II using standard protocols with a FastQ file produced from the SmartAnalysis pipeline. Subsequent typing results were generated using the proprietary HistoS and HistoTyper reporting softwares^50^. Sequence reads achieved a depth of at least 100× coverage of the targeted exons. A subset of 90 individuals from Uganda were also typed using Sanger-sequence-based HLA typing performed by an accredited tissue typing laboratory at Addenbrooke’s Hospital, Cambridge University Hospitals NHS Foundation Trust using the proprietary uTYPE software version 7 (Fisher Scientific). The possible ambiguous calls were minimized by using the ‘allele pair’ export function in this software, which lists all possible and permissible allele pair possibilities for each locus for each individual. Alleles were defined using the IMGT/HLA Release: 3.22.0 October 2015. Best-call allele pairs for each locus in each individual were determined based on local guidelines, prioritizing alleles that were ‘common and well-documented’^51^, but any genotype inconsistencies were highlighted and inspected manually for potential evidence of novel mutation. In a subset of the 1000Gp3 populations, allele calls were available from a previous round of lower resolution (four-digit or two-field) typing using Sanger sequencing^52^. These calls were used to test reliability of typing and estimate reductions in ambiguity calls for African, compared to Han Chinese, South China (CHS), and British from England and Scotland (GBR) individuals also from 1000 Genomes populations.

##### Early platform comparisons

A total of 47 unrelated individuals from Entebbe were used to validate the MiSeq generated calls by undertaking a comparison of Sanger-based and MiSeq-based typing. The Sanger-based typing method was undertaken using a moderately different set of exon coverage: exon 2 alone (*HLA-DRB3*, *HLA-DRB4* and *HLA-DRB5*); exons 2 and 3 (*HLA-DPA1*, *HLA-DQA1*, *HLA-DQB1* and *HLA-DRB1*); exons 2, 3 and 4 (*HLA-A* and *HLA-B*, *HLA-C* and *HLA-DPB1*). In all cases, any observed discrepancies could be resolved by taking into consideration differential exon coverage or the ability to resolve *cis*/*trans* ambiguities using the MiSeq platform. No discrepancies were observed due to differences in IMGT/HLA releases used for calling. The MiSeq platform was deemed superior for large-scale typing owing to the increased ability to resolve ambiguities.

HLA types were available for a subset of the 1000Gp3 samples from an earlier study using an older version of the IMGT/HLA release and older Sanger sequencing-based methods as described above. These data were used to demonstrate the utility of our methods compared to traditional methods through reducing ambiguous allele calls (Supplementary Fig. 1a).

A summary of the numbers of African individuals with data generated on each platform (MiSeq, PacBio, Sanger and intersecting array or NGS variant calling) is provided in Supplementary Table 6.

#### Novel *HLA* alleles

The novel alleles described below relate only to exon coding in African populations. These results are summarized in Supplementary Fig. 2a and Supplementary Table 7.

*HLA-A*

7 novel alleles were observed in 21 individuals across 7 populations of which 5 were identified using MiSeq (exons 1 and 4) and 2 were only captured using long-read sequencing (including exons 5 and 6).

*HLA-B*

2 novel alleles were observed in 2 individuals (one from ACB and one from Uganda) of which both were detected using MiSeq.

*HLA-C*

5 novel alleles were observed in 5 separate individuals spanning 4 populations; 4 of the novel alleles were detected using MiSeq and 1 using PacBio with novel variation in exon 6.

*HLA-DPA1*

14 novel alleles were identified in 54 individuals from all included populations except Burkina Faso; 10 of the novel alleles were detected using MiSeq and 4 were detected using PacBio with variants in exons 3, 4 and 5.

*HLA-DPB1*

8 novel alleles were identified in 12 individuals from 5 populations of which 5 were identified using MiSeq and 3 were detected with PacBio due to variation in exon 4.

*HLA-DQA1*

7 novel alleles were identified in 16 individuals from 4 populations of which 5 were detected using MiSeq and 2 were identified using PacBio due to variation in exon 4.

*HLA-DQB1*

6 novel alleles were identified in 16 individuals from 6 populations of which 4 were identified using MiSeq and 2 were identified using PacBio due to variation in exon 4.

*HLA-DRB1*

2 novel alleles were observed with one individual in each of ACB and South African populations of which both were detected using MiSeq.

#### Quantitative vaccine response antibody assays

Three validated multiplex immunoassays were used to measure antibody concentrations against a number of vaccine antigens in the three VaccGene populations. Briefly, this method measures total IgG against each respective antigen including functional (for example, neutralizing) as well as nonfunctional antibodies. Antibodies against DT, TT, PT, PRN, FHA and MV were determined in the MDTaP assay, which is a combination of two previously described assays^53,54^. Antibodies against Hib polysaccharide were determined in the HiB assay^55^. For MV and DT, the correlation of the multiplex immunoassay to gold-standard functional assays is high^56,57^. The immunoassay uses Luminex technology (Luminex) that depends on conjugation of commercially available or in-house developed antigens to fluorescent carboxylated beads using a two-step carbodiimide reaction to covalently link each antigen to a uniquely fluorescing bead. For the MDTaP assay, serum samples were diluted at concentrations of 1:200 and 1:4,000 in PBS/Tween-20/3% BSA and incubated with the beads to allow the binding of any antibody present in the medium while minimizing background in a manner similar to a monoplex solid-phase ELISA. The bead–antigen–antibody complexes were then separated from remaining plasma or serum using a vacuum manifold before washing with PBS and incubating with a further anti-human IgG antibody conjugated to R-phycoerythrin sourced from Jackson ImmunoResearch Laboratories, and washing again before detection in the Luminex flow cytometer. The HiB assay was performed similarly, with the exception that samples were diluted at a concentration of 1:100 in 50% antibody-depleted human serum. The cytometer was used to firstly detect the identity of the fluorescently labeled bead (and therefore antigen bound), and then secondly to detect the fluorescence intensity of R-phycoerythrin (related to the concentration of primary antibody in solution) bound to each bead passing through the detection channel^54^. The final concentration of bound antibody was calculated by determining the median fluorescence intensity of the antigen-specific beads and using diluted standards to calculate the concentration in international units for each antigen. ELISA results were available for MV vaccine and TT antibody responses from a subset of the Entebbe participants as performed as part of the early investigation undertaken in the Ugandan cohort^42^. HBsAg responses were measured using the anti-HBs kit on the ABBOTT Architect i2000 using recommended protocols (Abbott Laboratories). HBsAg measures had an upper limit of detection at 1,000 mIU ml^−1^. Final antibody measures were saved and linked with demographic data using Microsoft Excel 2016 (16.0.5435.100).

#### Genome-wide genotyping

SNV genotyping was undertaken for the three VaccGene populations using the Illumina HumanOmni 2.5 M-8 (‘octo’) BeadChip array version 1.1 (Illumina), performed by the Genotyping Core facilities at the Wellcome Sanger Institute. Genomic DNA underwent whole-genome amplification and fragmentation before hybridization to locus-specific oligonucleotides bound to 3-μm-diameter silica beads. Fragments were extended by single base extension to interrogate the variant by incorporating a labeled nucleotide enabling a two-color detection (Illumina, 2013). Genotypes were called from intensities using two clustering algorithms (Illuminus and GenCall) in GenomeStudio (Illumina) incorporating data from proprietary predetermined genotypes.

#### Whole-genome sequencing of MKK

Whole-genome sequencing to a 30× coverage was undertaken for the MKK using the Illumina HiSeq X platform using a PCR-free library preparation with a PhiX control spike-in on a barcoded tag. Basecalling was performed on the instrument by using Illumina’s sequencing control software (SCS version 3.3.76) and the real-time analysis software. The resulting basecalls were converted directly to unmapped BAM format using the Wellcome Sanger Institute’s BAMBI software (version 0.9.4) for injection into our mapping pipeline. The mapping pipeline first removes any adaptor sequence from the SEQ portion of the read and annotates it as an AUX tag to be replaced in the SEQ after mapping as a soft-clipped sequence. A spatial filter was next generated for the lane to remove any bubble-induced artifacts from the flowcell by mapping the PhiX sequence to the reference using BWA MEM (version 0.7.15-r1140) and using this to create a mask to remove any contiguous blocks of spatially oriented INDELs using our spatial filter program (pb_calibration version 10.27) after alignment. Meanwhile, the human data were mapped to HS38dh using BWA MEM (version 0.7.15-r1140). The output from this process was then converted from SAM to BAM using scramble (version 1.14.8); headers were corrected using samtools reheader (version 1.3.1-npg-Sep2016); and then the data were sorted and had duplicates marked using biobambam (version 2.0.65). Any stray PhiX reads were removed using AlignmentFilter (version 1.19) and the resulting CRAM file was delivered to the Sanger core IRODS facility for storage and transfer to The European Genome-phenome Archive.

Single-sample variant calling to genomic variant call format (GVCF) was performed using GATK HaplotypeCaller (version 3.8-0-ge9d806836). GVCF files were combined into a single GVCF file using GATK CombineGVCFs (version 2017-11-07-g45c474f), and then the final VCF callset was created using GATK GenotypeGVCFs and genomic coordinates lifted over to build 37 using LiftOver (https://genome.sph.umich.edu/wiki/LiftOver).

#### RNA-seq of 1000Gp3 LCLs

A custom RNA-seq read alignment approach was used to identify eQTL for the *HLA* genes. The HLA region presents a major challenge in determining RNA-seq-based gene expression quantification due to the abundance of paralog sequences that are highly polymorphic. We therefore aligned the short RNA-seq reads to a reference sequence defined per individual, complemented with alternative *HLA* alleles to improve the mapping of the reads. The eQTL analysis involved the quantification of expression of the following nine *HLA* genes: *HLA-A*, *HLA-B*, *HLA-C*, *HLA-DQA1*, *HLA-DQB1*, *HLA-DPA1*, *HLA-DPB1*, *HLA-DRB1* and *HLA-DRB5*.

RNA-seq was undertaken using existing LCLs from 600 unrelated samples from five African populations in the 1000 Genomes Project, including the 97 LWK, 84 MSL, 112 GWD, 99 ESN, 42 YRI from 1000Gp3 as well as 166 MKK from the HapMap project. Cell lines were retrieved from Coriell in preassigned batches. To reduce batch effects, the samples were divided into batches for sequencing representative of all six populations. Cell cultures were expanded, and 1 × 10^7^ cells per line were pelleted, treated with RNAProtect (Qiagen) and stored at −80 °C until shipment. Following further randomization, RNA extraction from the entire pellets was performed by Hologic/Tepnel Pharma Services using the RNeasy PLUS mini kit (Qiagen). Library preparation was then performed using the standard automated Kapa stranded mRNA library preparation protocol, followed by RNA-seq on the HiSeq 2500 using paired-end sequencing with 75-bp reads. The sequencing was carried out at the Wellcome Sanger Institute where 12 samples, randomized across populations, Coriell batches and Hologic RNA extraction batches, were sequenced over two lanes to ensure adequate coverage to quantify gene expression while minimizing systematic bias.

#### Follicular helper T cell assay

An AIM method was used to measure and compare proportions of circulating antigen-specific T_FH_ cells in the circulating blood of donors defined by *HLA-DRB1* allele carriage. The AIM assay uses flow cytometry to detect proportions of antigen-specific follicular helper T (T_FH_) cells defined as coexpressing CD25, OX40 and CXCR5 markers following ex vivo antigen stimulation of peripheral blood mononuclear cells^24^. Based on the *HLA-DRB1* allele type, 1 × 10^6^ peripheral blood mononuclear cells were selected from stored samples collected from consenting participants recruited into studies coordinated by the laboratory of A.S. investigating immunodominant peptides associated with responses against pertussis^58^, tuberculosis^59^, dengue^60^ and IgE allergy^61^. The samples were thawed and cultured with 30 μg ml^−1^ PT (Reagent proteins), 5 μg ml^−1^ DT (Reagent proteins), 5 μg ml^−1^ TT (List Biological Laboratories), 10 μg ml^−1^ phytohemagglutinin (Sigma) or toxoid diluent (water) at 37 °C for 24 h. The cells were then washed, labeled with an antibody panel for 15 min at 4 °C before being fixed with paraformaldehyde (Sigma) and acquired on an LSR II (Becton, Dickinson and Company). The antibody panel was as follows: CCR7-PerCP-Cy5.5 (G043H7), OX40-PE-Cy7 (BerACT35), CXCR5-Brilliant Violet 605 (J252D4), all from BioLegend; CD45RA-eFluor450 (HI100), CD4-APC-eFluor780 (RPA-T4), from eBioscience; CD25-FITC (M-A251), CD14-V500 (M5E2), CD19-V500 (HIB19), CD8-V500 (RPA-T8), from BD Biosciences; and LIVE/DEAD Aqua stain (Thermo Fisher Scientific). Data derived from the gating strategy were analyzed using FlowJo Software version 10 and either one-tailed Wilcoxon rank-sum tests or linear regression statistical tests were performed in R. All participating donors were known either to have received DT and TT, and either whole-cell pertussis (wP together known as DTwP) or acellular pertussis (aP, together as DTaP) as part of a vaccine study undertaken in the laboratory of A.S., or self-reported as having received standard vaccines during childhood.

#### Cell-specific HLA-wide eQTL analyses

HLA typing was performed on DNA extracted from 91 individuals as part of the Database of Immune Cell eQTLs (DICE) dataset^62^ using the same Histogenetics MiSeq protocol described above.

### Analytical methods

#### SNV QC

SNV QC was performed separately for each genotyped VaccGene cohort using identical steps and using SNVs mapped to Human Genome Build 37. Low-quality variants that mapped to multiple regions within the human genome or did not map to any region were removed. Samples with a call rate of less than 97% and heterozygosity greater than three standard deviations around the mean were filtered sequentially. Sex check was performed in PLINK (v1.7) using default *F* values < 0.2 for males and > 0.8 for females. Samples with discordance between reported and genetic sex were removed. Genetic variant filtering was performed across the remaining samples, and sites called in <97% samples were removed from each population. Identity by descent (IBD) was measured within each population. Only samples with IBD > 0.9 not known to be twins were removed using a custom algorithm that removed the sample from the pair with the lower variant call rate. Sites in Hardy–Weinberg disequilibrium (*P* < 10^−^^8^) were also excluded from future analysis in all individuals, calculated using individuals with IBD < 0.05 (hereafter, designated ‘founders’). Following the above QC steps, principal component analysis (PCA) was performed in EIGENSOFT (v4.2)^63^ for each population and combined with populations representative of other parts of Africa (the ‘AGV dataset’^20,64,65^) or global populations including 1000 Genomes (‘Global + AGV dataset’). PCA was carried out after LD pruning to a threshold of *r*^2 ^= 0.5 using a sliding window approach with a window size of 50 SNVs sliding 5 SNVs sequentially. Regions of long-range LD were removed from the analysis. Individuals with values of the first 10 principal components more than six standard deviations around the mean of other samples in each population were removed.

#### Genotype imputation

Haplotype phasing was undertaken in each VaccGene population separately using SHAPEIT2 (ref ^66^; v2.r790) with standard parameters and the advised effective population size of 17,469. We subsequently used IMPUTE2 (v2.3.2) to estimate unobserved genotypes using a combined reference panel consisting of the 1000 Gp3 reference panel combined with data from the African Genomes Variation Project^20^ and a 4× whole-genome sequence coverage dataset of another Ugandan population of 2,000 individuals entitled the UG2G dataset: 1000G/AGVP/UG2G^20^.

#### Cohort genotype variant merging

A high-quality set of autosomal genotype calls free of batch effects were required for a number of downstream analyses. Variant calls derived from a combination of array genotyping (Illumina Omni 2.5 M passing QC in the VaccGene and some 1000Gp3 cohorts) and NGS for other 1000Gp3 populations (using only calls at sites intersecting with Omni 2.5 M typed locations) were defined. A comparison of variant calls between array and NGS platforms was undertaken for a subset of 1000Gp3 individuals who had data from both platforms using concordance. Only those sites with concordance estimates of *r*^2^ > 0.99 were taken forwards for further analyses. Variants typed on the Omni 2.5 M array were called in all individuals using array genotypes as first priority (where data were available from both array and NGS platforms) and then using NGS data (if array data were not available). Once variant calls were available for all individuals, these variants were used to calculate principal components and ADMIXTURE (v1.2) analysis across all autosomes to ensure that there was minimal evidence of batch variation caused by a differential use of NGS or array variants across individuals and populations.

#### Measuring differentiation of *HLA* alleles across African and global populations

G_ST_ was calculated for each locus using alleles described in two-digit, four-digit and six-digit resolution using the ‘diveRsity’ package in R^67^. G_ST_ and Jost’s *D* statistic are statistics explicitly designed for multi-allelic residues. Both statistics were calculated but, given the close correlation between the two outputs, the availability of G_ST_ statistics in other studies of HLA in Africa^68^ made this the statistic of choice. Allelic richness was calculated in diveRsity using bootstrap sampling (1000 samples) with replacement to estimate the average number of alleles observed with standard errors given the differing number of individuals observed in each population and the likelihood of observing rare alleles.

#### Vaccine antibody response normalization

Measured antibody responses were normalized using both logarithmic and inverse normalization of traits in R version 3.5.1. Inverse-normalized traits were tested for association with a variety of available metadata endpoints to determine covariates to include in the final regression model to increase power in the quantitative analysis^69^. Endpoints included time between vaccination and sampling, sex, age, weight-for-length *z*-score at birth, number of illnesses, socioeconomic status and HIV status (if known). Only time between vaccination and sampling was used in the final models. Inverse normal transformation measures were used throughout our analyses and all results are reported as such, unless stated otherwise.

#### Intra-cohort genotype association testing and meta-analysis

Multiple software packages are available that can account for population structure and cryptic relatedness in genomic association studies using mixed-model approaches^70^. However, until recently only a handful of these algorithms could simultaneously account for probabilities of imputation accuracy in large datasets. We therefore applied a mixed model in our association analyses implemented in the GEMMA (v0.94) software^71^ that explicitly accounts for imputed genotypes. We calculated the relatedness matrices using only those autosomal variants directly typed in each population. Inclusion of the first ten principal components did not affect the association statistics for any tested phenotype in any cohort as would be expected given that these models explicitly account for population structure and relatedness and so these PCs were not included in any downstream association testing. The METASOFT (v1.0) software was used to undertake fixed and random-effect meta-analysis to test for shared signals of association across populations^72^.

#### HLA imputation and HLA reference panel construction

The HLA*IMP:02 software was used for imputing classical *HLA* alleles to two-digit and four-digit resolution at all 11 loci in VaccGene individuals with available genotype data^21^. HLA*IMP:02 was used preferentially above other software including SNP2HLA^73^ and HIBAG^74^ because of the inclusion of individuals of West African ancestry in the reference panel of HLA*IMP:02 and the reported accuracies of imputation of individuals from diverse population backgrounds^21^ making this algorithm a natural choice. Furthermore, the explicit handling of missingness of types between individuals and the adaptability of the algorithm by our team to allow for higher-resolution types and amino acid imputation allowed a more straightforward implementation. Imputation of *HLA* alleles in the African and UK (ALSPAC) populations was performed (a) using the March 2016 release of the HLA*IMP:02 reference panel with default settings to establish a baseline for accuracy and (b) using an African-specific reference panel with algorithmic modifications, described below. The ‘best-guess’ call was defined for each diploid allele in every individual using the output from the algorithm in the presence or absence of an imposed threshold for calling using the posterior probability of 0.7. It has been proposed that imposing this threshold improves the quality of the total number of calls at the expense of reducing the total number of available calls. In downstream association analyses, this posterior probability was used as variant dosages to account for probabilities in regression analyses.

The African-specific reference panel was built using only variants (derived from publicly available array genotype or whole-genome sequencing data for 1000Gp3 and MKK populations or array genotypes for the VaccGene populations as described above) and six-digit ‘G’ calls from the 1,705 typed individuals calling any novel alleles as missing. Fivefold cross-validation, comprising five random splits of the reference dataset into training (four-fifths of the data) and validation (one-fifth of the data) sets, was carried out to evaluate expected imputation accuracy on African samples. For each split, accuracy in the validation set was assessed using the metrics described below. All imputations used for association analyses were based on the complete reference panel.

Comparisons between imputed versus typed calls were undertaken at the four-digit (that is, two-field) level of resolution. If an available call at a single allele locus included several potential higher-resolution alleles (that is, a list of potential ambiguities), only the first available allele calls from either platform (adhering to a ‘common and well-documented’ priority) were used for comparison. In the cases of comparing imputed HLA calls to typed calls, any six-digit ‘G’ type calls were reduced to four-digit ones and treated as the ‘truth’ set. By comparing each individual allele in turn, it was possible to define calls of the test platform that were:

- True positives (TPs)
- False positives (FPs); called by the test platform as that allele when it was in fact another allele according to the truth)
- False negatives (FNs; called by the test platform as another allele when it was in fact this allele)
- True negatives (TNs).

Thus, at the level of an individual allele, various metrics could be calculated. Sensitivity was defined as:

TP / (TP + FN)

Specificity was defined as:

TN / (TN + FP)

Positive predictive value was defined as:

TP / (TP + FP)

Negative predictive value was defined as:

TN / (TN + FN)

Accuracy was defined as:

(TP + TN)/(TP + FP + FN + TN*)*

Concordance was calculated at the level of the locus. For every pair of chromosomes with data available in both truth and test sets, the number of identical allele calls between platforms was calculated and divided by the total number of alleles, equivalent to the positive predictive value. Any individuals with missing alleles on either or both chromosomes on either platform were excluded from these calculations.

HLA imputation using the broad multiethnic panel was performed using the multiethnic HLA reference panel (version 1.0 2021) available on the Michigan imputation server using recommended settings^22^.

#### Pooled linear mixed-model and HLA variant association testing

To undertake conditional analyses including all genotyped and imputed genotype variants across the *HLA* locus in addition to *HLA* allele and amino acid variants across all three populations, we leveraged the intra-cohort normalized, quantitative nature of the antibody responses and combined all individual-level genetic data from individuals in all three VaccGene populations maintaining imputation dosages where appropriate. For *HLA* alleles and amino acids, posterior probabilities were used to infer imputation dosages at each allele. We calculated a relatedness matrix using only directly genotyped autosomal variants from the three populations, and we then undertook association testing using dosages in GEMMA (v0.94) to account for imputation probabilities in the context of both imputed genotypes and *HLA* alleles and amino acid variants. The resultant *P*-value association statistics were then compared to output from the fixed-effects meta-analysis approach determined using METASOFT (v1.0) using the Pearson correlation coefficient. Stepwise forward conditional modeling was used for each trait including the index SNV dosages as fixed-effect covariates in the model to assess for evidence of interdependence while taking differential LD patterns into account across all populations.

#### Fine-mapping HLA associations with each trait

An approach similar to that used by Moutsianas and colleagues investigating the effect of HLA in multiple sclerosis^17^ was used to compare and contrast the results of both manual and automated stepwise linear modeling approaches. First, stepwise conditional modeling was performed using the phylogenetic linear mixed-model approach in GEMMA (v0.94) for each trait to identify independently associated loci achieving a significance threshold of *P* ≤ 5 × 10^−^^9^. This approach resulted in a range of SNVs, *HLA* alleles or amino acids likely to be independently associated with each trait, frequently spanning multiple loci across the class II region. The gene origins of these ‘independent index’ variants were determined (SNV or amino acid residues in HLA-DRB1, for example), and the dosages of all variants were then incorporated in a manual modeling approach. For this manual approach, a refined number of unrelated individuals (IBD < 0.2) were selected, and models of association were tested using additive dosage probabilities for imputed genotype, classical allele and biallelic amino acid residues across all 11 loci with a population-average minor allele frequency greater than 0.01. Null models were defined for each trait by including the first five genetic principal components and the ‘time between sampling most recent vaccination’ covariate. Independent index variants discovered through the phylogenetic linear mixed-model analyses were assessed both in univariate models (that is, single SNV, *HLA* allele or biallelic amino acid residue variable) or multivariable models (that is, defining more than one single SNV, HLA allele or amino acid residue). Models were rationally tested and compared based on the known associations between amino acid residues and classical alleles. For example, an arginine at position 74 in the HLA-DRB1 protein (designated DRB1-74Arg) is only found in alleles in the two-digit HLA-DRB1*03 allele group. Using the six-digit ‘G’ resolution, the only allele groups therefore containing DRB1-74Arg include HLA-DRB1*03:02:01 and HLA-DRB1*03:01:01 G. Each model defined using this framework was tested and compared. Using the given example, univariate models comparing the DRB1-74Arg and HLA-DRB1*03 variants, and a conditional model including both HLA-DRB1*03:02:01 and HLA-DRB1*03:01:01 G would be compared. All models included the same principal components and time covariates as defined in the null model for each trait. The models were compared to the null using the likelihood ratio test, if the models were nested, or using the BIC otherwise. Models with lower BIC values were interpreted to explain the variance in the observed data most parsimoniously.

Finally, any prior knowledge from the associations derived from the linear mixed-model associations were removed, and automated bidirectional stepwise model selection based on the BIC was undertaken. This modeling was designed to test whether models incorporating amino acid residues or classical alleles best explained each trait at each locus and also to determine whether any other variants should be considered in a final model other than those identified using the manual approach above. A consensus model was then determined based on the results of the manual and automated approaches for each trait. Manual and automated modeling steps were performed in R 3.5.1.

#### RNA-seq and eQTL analysis

RNA-seq reads were inspected using the FastQC tool for QC. Reads were trimmed using Cutadapt for polyA and adaptors before mapping. The merged set of whole-genome genotypes derived from a combination of array and sequencing data from VaccGene, 1000Gp3 and HapMap samples were used for the eQTL data analysis. All samples with RNA-seq data available also had genotype data available. Variant calls from both genotype and sequencing data for these samples were included in eQTL analyses. After accounting for QC of the RNA-seq data, 558 samples were available for the eQTL analysis: ESN (99), GWD (112), LWK (97), MKK (126), MSL (83) and YRI (41).

The RNA-seq dataset was mapped to a custom genome reference sequence that consisted of the non-HLA-containing human reference sequence (hg38) and HLA-containing reference sequence unique to each individual. The HLA-containing reference was generated based on the six-digit ‘G’ type results of the samples in our dataset. We extracted a total of 285 HLA alleles: 47 *HLA-A*, 73 *HLA-B*, 35 *HLA-C*, 11 *HLA-DPA1*, 39 *HLA-DPB1*, 8 *HLA-DQA1*, 25 *HLA-DQB1*, 45 *HLA-DRB1* and 2 *DRB5* nucleotide sequences of exons from the international ImMunoGeneTics/HLA database v3.33.0 at the European Bioinformatics Institute. For each *HLA* allele, we generated a sequence where the exons of the respective allele were merged with 200 bases of spacers (N) as introns. The exons that were not typed in the ImMunoGeneTics/HLA database for each *HLA* allele were filled using the closest allele. The resulting HLA-containing reference contained 285 *HLA* gene structures with the corresponding exons and the introns of N characters. We generated an annotation file for the HLA-containing reference in the form of a GTF file as well as the exon–exon junction file for the mapping. The on-HLA-containing reference was generated from the human reference sequence (hg38) excluding the alternative haplotype contigs where the nine HLA genes in the reference were removed from the reference sequence by hard masking. We used the corresponding Ensemble gene annotation (v83) for the non-HLA reference sequence. The custom reference sequence for the RNA-seq data mapping was generated by merging the non-HLA-containing reference sequences with the HLA-containing reference sequences. The annotations and the exon–exon junctions were merged to generate the final gene annotation GTF file for the mapping.

Alignment was performed using the STAR alignment tool^75^ in two-pass mode. Our custom reference sequence and the custom gene annotations were used for the indexing of the reference sequence for the mapping. During the second pass, we used the novel exon–exon junctions as well as the exon–exon junctions we generated for the HLA-containing reference. The quantification of RNA transcripts was strongly affected by reads that mapped to multiple locations in the custom reference sequence. Since we had 285 *HLA* alleles with high similarity in our reference and the default maximum number of multiple alignments in STAR aligner is 10, we increased the maximum number of multiple alignments to 300 for the RNA-seq mapping. We counted the number of reads mapping to the HLA haplotypes using a custom method using the htslib for accessing the alignment files in bam format. We used two criteria to count the reads: (1) If the reads were mapped to the multiple HLA haplotypes, but no other regions in the genome, we counted these reads as single mapping; (2) If the reads were mapped to a unique *HLA* allele, the reads were counted for that allele. After verifying the reads were mapping to their correctly typed *HLA* alleles, we quantified the gene expression for each HLA gene as the sum of these counts. The read counts for the other genes were calculated with htseq-count v0.9.1, using the gene annotations from Ensembl as the features. The counts were merged to include the whole set of gene counts. Normalization was performed using the DESeq2 tool with the variance-stabilized transformation^76^. The variance-stabilized transformation was performed after the library size and dispersion estimation. Normalization was performed for each population separately.

eQTL mapping was performed for the 5-Mb region that included the nine HLA genes of interest. We restricted our search to *cis*-eQTLs by selecting variants within 1 Mb of each gene’s start and end positions. Per population, *cis*-eQTLs were identified by linear regression where normalized gene expression was regressed on variant dosage correcting for covariates using Matrix eQTL^77^. Covariates included population principal components calculated from genotype data, metadata on known technical variables and unobserved confounding variables detected using surrogate variable analysis. Per population for each variant, we calculated the *P* values that are corrected using the Benjamini–Hochberg procedure and the beta values. The results of the eQTL analysis for six populations were then combined using a fixed-effects model implemented by METASOFT (v1.0).

The same methods were used for the individual cell types using the DICE dataset. This dataset included 14 cell types.

To test the reproducibility of our approach, we replicated a well-characterized eQTL for HLA-C associated with differential control of HIV-1 (ref. ^35^) in the 1000Gp3 dataset. We observed a strong effect of rs2395471 on HLA-C expression in the African populations (*P* = 1.14 × 10^−^^12^) in the same direction as reported previously. We also replicated a signal reported recently for variable *HLA-DRB1* expression where the A allele of rs9271108 was associated with an increased expression of *HLA-DRB1* (ref. ^78^). We observed the same direction of effect (beta 0.32, *P* = 1.6 × 10^−^^10^) in our tested African populations.

#### Trait and genetic correlation

Correlation between normally distributed continuous variables or traits were tested using Pearson’s correlation coefficient. Equivalent testing for variables or traits not considered continuous or sufficiently normally distributed was undertaken using Spearman rank. Testing for the significance of correlation between HLA amino acid residues derived from the present study and a historical GWAS of self-reported pertussis^23^ was performed using permutation. The null distribution was calculated by randomly assigning different SNV identities to the calculated beta coefficients from the pertussis GWAS and recalculating Pearson’s *r* between 100,000 to 100,000,000 times (dependent on whether a *P* value could reliably be calculated). The *P*_perm_ value was calculated as the frequency at which a Pearson’s *r* value calculated from permutation was observed to surpass the *r* value from the true data. These calculations were undertaken using both complete variant datasets and datasets pruned by LD (keeping only the top associated SNV and those SNVs with *r*^2^ < 0.35).

#### Peptide binding assays

The IEDB^79^ was used to test whether the affinity or breadth of peptides derived from specific protein sequences differed by groups of *HLA* alleles defined as being associated with increased or decreased antibody responses. The output from the binding prediction algorithm included a binding affinity prediction (IC_50_, in nM) and a percentile rank generated by comparing the predicted IC_50_ against scores of 5,000,000 random 15-mers selected from the SWISSPROT database^80^. The percentile rank scores of 15-mer peptides derived from PT (GenBank accession ALH76457), DT (BAL14546) and TT (WP_011100836) were compared. The highest affinity peptide per protein and allele was defined using the peptide with the lowest percentile score. To increase power to identify differences between groups of alleles, all *HLA-DRB1* alleles present in the IMGT database were divided into groups dependent on their sequences and whether they possessed an excess of residues associated with either increased (defined as ‘*DRB1-233Thr*’ alleles for PT) or decreased (defined as ‘*DRB1-233Arg*’ alleles) antibody responses. The definition of these alleles for PT vaccine responses was undertaken as follows. Firstly, the number of residue positions found to be significantly (*P* < 0.05) associated with either PT (*n* = 39) response was determined, and then alleles were defined as to whether they had an excess (>1.5×) of residues associated with either a positive beta or an excess ( > 1.5x) of negative beta effect estimates. The distributions of affinities of the top-predicted binding peptides for each of the alleles classified as such were then compared and tested for differences using a two-tailed Mann–Whitney *U* test. The breadth of antigen-specific peptide binding by class II *HLA* alleles was defined by measuring the proportion of peptides predicted to bind within the top 5th percentile of all peptides from each peptide per allele of interest, compared across antigens and allele groups.

### Ethics and inclusion statement

This study was explicitly designed to understand the factors influencing diverse response to vaccination in low- and middle-income countries and in line with this design, the main data used for the analysis were gathered from cohorts of infants across three diverse sites across Africa (Uganda, South Africa and Burkina Faso) defined as part of the VaccGene consortium. Local scientists from each site were involved in every stage of the study including design, cohort recruitment and re-engagement for genetics studies, sampling, sample handling and data acquisition and, where possible, analysis. For example, A.M. from Uganda was involved in sample selection, DNA extraction, QC and preparation for genotyping and received training in bioinformatics analysis. D.K. worked closely with P.K. and A.M.E. and alongside A.J.M. in Uganda to design re-consenting protocols to ensure recruited participants were informed about potential genetic analyses and organized participant meetings to discuss the design and preliminary results from the study. C.C. and S.A.M. from South Africa, and A.D. and S.S. from Burkina Faso were involved in the early design and sample and data collection from the replication cohorts in their respective countries. A.D. and C.C. were critically involved in sample preparation. S.S., S.A.M. and A.M.E. are the custodians of the data from Burkina Faso, South Africa and Uganda, respectively. Collectively, the collaboration has already facilitated numerous independent research outputs using the derived genetic data for researchers from the original LMIC settings. As such, we fully endorse the Nature Portfolio journals’ guidance on LMIC authorship and inclusion.

This research is locally relevant to all studied countries given that it provides findings on genetics and other sociodemographic factors affecting vaccine response and provides a series of resources that may be useful for future research into this area.

All of the research was approved by ethical review boards both locally in the country of focus, and within the United Kingdom. The data collection and analysis techniques used raised no risks pertaining to stigmatization, incrimination, discrimination, animal welfare, the environment, health, safety, security or other personal risks. Derivatives of blood samples were transferred out of the countries for antibody assays or genotyping but have since been either destroyed or transferred back to their original country of origin. No other biological materials, cultural artifacts or associated traditional knowledge has been transferred out of any country. In preparing the manuscript, the authors reviewed relevant studies from each of the countries involved in this study.

### Reporting summary

Further information on research design is available in the Nature Portfolio Reporting Summary linked to this article.

## Online content

Any methods, additional references, Nature Portfolio reporting summaries, source data, extended data, supplementary information, acknowledgements, peer review information; details of author contributions and competing interests; and statements of data and code availability are available at 10.1038/s41591-024-02944-5.

### Supplementary information

Supplementary Information Supplementary Figs. 1–4, Supplementary Tables 1–3, 5–7, 11, 12, 14–16 and 18 and descriptions of Supplementary Tables 4, 8–10, 13 and 17.

Reporting Summary

Supplementary Table 4 Association statistics from the fixed-effects meta-analyses for all extended MHC variants (biallelic single-nucleotide polymorphisms, HLA alleles and amino acids) in the three African populations for the five vaccine antibody responses with GWAS significant associations. The original results were derived from the pooled linear mixed model described in the main text. Single-nucleotide polymorphisms are coded in build 37 coordinates as ‘chromosome’:’base pair’:’minor allele’:’major allele’. Amino acids are coded as ‘Gene’_’AA1’_’full length position’_’amino acid present’_’coding sequence position’. Coding sequence position was used in the main text. HLA alleles are coded as ‘Gene’_’6 digit G Allele’.

Supplementary Table 8 Allele-specific statistics comparing imputed HLA allele calls from HLA*IMP:02 to sequence-based six-digit ‘G’ typing divided by population. Calls are compared at four-digit level of resolution and presented in four-digit format. Locus A and allele 0101 refers to HLA-A*01:01, for example. New alleles defined through HLA typing are donated as XX and are detailed in Supplementary Table 7.

Supplementary Table 9 Allele-specific statistics comparing imputed HLA allele calls from HLA*IMP:02G to sequence-based 6-digit ‘G’ typing divided by VaccGene population. Calls are compared at four-digit level of resolution and presented in four-digit format for comparison to Supplementary Table 8. Locus A and allele 0101 refers to HLA-A*01:01, for example. New alleles defined through HLA typing are donated as XX and are detailed in Supplementary Table 7.

Supplementary Table 10 Allele-specific statistics comparing imputed HLA allele calls from HLA*IMP:02G to imputed HLA allele calls from the broad multiethnic reference panel divided by VaccGene population. Calls are compared at four-digit level of resolution and presented in four-digit format for comparison to Supplementary Table 9. Locus A and allele 0101 refers to HLA-A*01:01, for example. New alleles defined through HLA typing are donated as XX and are detailed in Supplementary Table 7.

Supplementary Table 13 Allele dosages and normalized antibody distributions for the 13 variants identified to be significantly associated with at least one antibody distribution from the imputation and fine-mapping exercise. Other relevant covariates including sex, genetic principal components 1–5 and time between last vaccine and sampling are all also provided. Data are available for the 2,411 individuals with IBD < 0.2, thus not requiring the genetic relatedness matrix or a mixed model to test for association.

Supplementary Table 17 Summary beta, standard error and *P* values for fixed-effects meta-analysis of *cis*-QTL analyses for each of eight major class I and II HLA genes. The original statistics were calculated using linear regression of the derived gene expression level in each individual population.

## Data Availability

All direct genotypes from VaccGene individuals after QC alongside imputed data and raw and curated HLA sequencing data and calls have been submitted to the European Genome-Phenome Archive under accession EGAS00001000918, with the datasets under EGAD00010002578 and EGAD00010002583 (Uganda); EGAD00010002582 and EGAD00010002580 (South Africa); EGAD00010002581 and EGAD00010002579 (Burkina Faso). The merged SNV calls for the African populations and the HLA allele calls and related sequencing data are found under EGAD00010002577 and EGAD00001011379, respectively. Data are available to researchers following application to the Wellcome Sanger Institute Data Sharing team (datasharing@sanger.ac.uk with details available at https://edam.sanger.ac.uk/) and review of an application by a Data Access Committee. The Committee are committed to rapid decision-making and ready access to data and will endeavor to decide on any requests received within 2 weeks of receipt to the Committee. Summary statistics for the genome-wide association tests of imputed data for eight vaccine antibody levels are available on Zenodo at 10.5281/zenodo.7357687 (ref. ^81^). The RNA-seq data for 1000 Genomes are available at ENCODE via https://www.encodeproject.org/search/?searchTerm=AFGR&type=Experiment, and data for DICE are available from https://dice-database.org/downloads/. HLA peptide binding data were derived from the IEDB accessed in 2017 and 2018, and the antigen sequences were downloaded from SWISSPROT.

## Code availability

The R script for calculating correlation in effect estimates for HLA amino acid residues comparing VaccGene pertussis antibody levels, and self-reported whooping cough from ALSPAC, is available on Zenodo at 10.5281/zenodo.10728920 (ref. ^82^). All other analyses used preassigned code defined within software packages that are publicly available as described in Methods. Any other requests for clarifications may be sought from the corresponding authors.
