# Supplementary material for: High-resolution African HLA resource uncovers HLA-DRB1 expression effects underlying vaccine response
Source: Nat Med. 2024 May 13;30(5):1384–94. doi: 10.1038/s41591-024-02944-5 (PMC11108778; doi:10.1038/s41591-024-02944-5)
Supplement: Supplementary file 1 — Supplementary Figs. 1–4, Supplementary Tables 1–3, 5–7, 11, 12, 14–16 and 18 and descriptions of Supplementary Tables 4, 8–10, 13 and 17. [file 41591_2024_2944_MOESM1_ESM.pdf]

nature medicine

Article https://doi.org/10.1038/s41591-024-02944-5

High-resolution African HLA resource uncovers *HLA-DRB1* expression effects underlying vaccine response

In the format provided by the
authors and unedited

Supplementary Materials for

High-resolution African HLA resource uncovers *HLA-DRB1* expression effects underlying vaccine response

Alexander J. Mentzer*, Alexander T. Dilthey, Martin Pollard, Deepti Gurdasani, Emre Karakoc, Tommy Carstensen, Allan Muhwezi, Clare Cutland, Amidou Diarra, Ricardo da Silva Antunes, Sinu Paul, Gaby Smits, Susan Wareing, HwaRan Kim, Cristina Pomilla, Amanda Y. Chong, Debora Y.C. Brandt, Rasmus Nielsen, Samuel Neaves, Nicolas Timpson, Austin Crinklaw, Cecilia S. Lindestam Arlehamn, Anna Rautanen, Denison Kizito, Tom Parks, Kathryn Auckland, Kate E. Elliott, Tara Mills, Katie Ewer, Nick Edwards, Segun Fatumo, Emily Webb. Sarah Peacock, Katie Jeffery, Fiona R.M. van der Klis, Pontiano Kaleebu, Pandurangan Vijayanand, Bjorn Peters, Alessandro Sette, Nezih Cereb, Sodiomon Sirima, Shabir Madhi, Alison M. Elliott, Gil McVean, Adrian V.S. Hill†, Manjinder S. Sandhu†*

*Corresponding authors. Email: Alexander J Mentzer alexander.mentzer@ndm.ox.ac.uk and Manjinder S Sandhu m.sandhu@imperial.ac.uk

†These authors contributed equally to this work

This PDF file includes:

Supplementary Figs. 1 to 4
Supplementary Tables 1 to 18

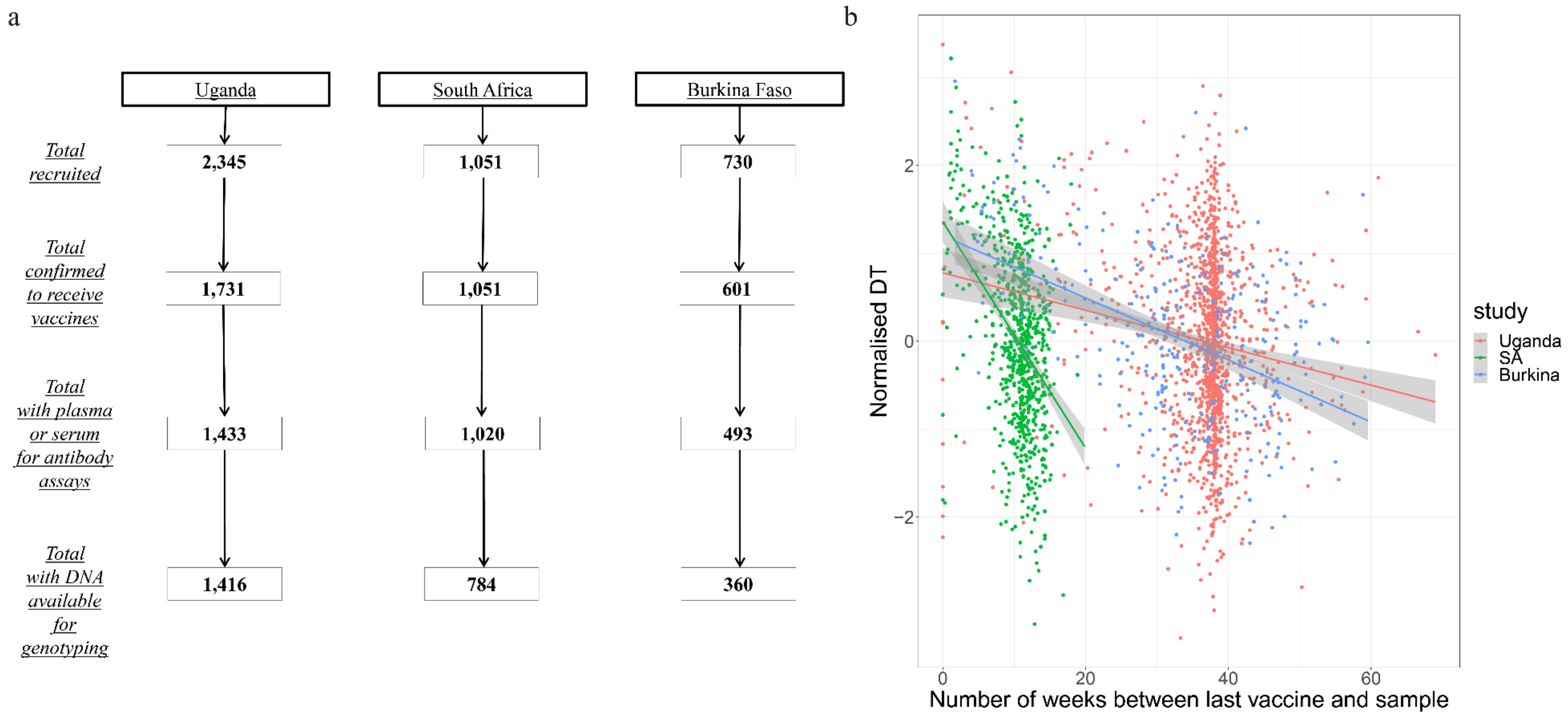

Supplementary Fig. 1.

A multi-population genome-wide association study of responses to eight vaccine antigens. (a) Individuals recruited with vaccine-related data and samples available for analysis as part of *VaccGene* in each cohort. **(b)** Time between final vaccination dose of DT and sampling for measuring response to DT within each population. The center lines are the linear lines of best fit colored by each population with shaded 95% confidence intervals**.**

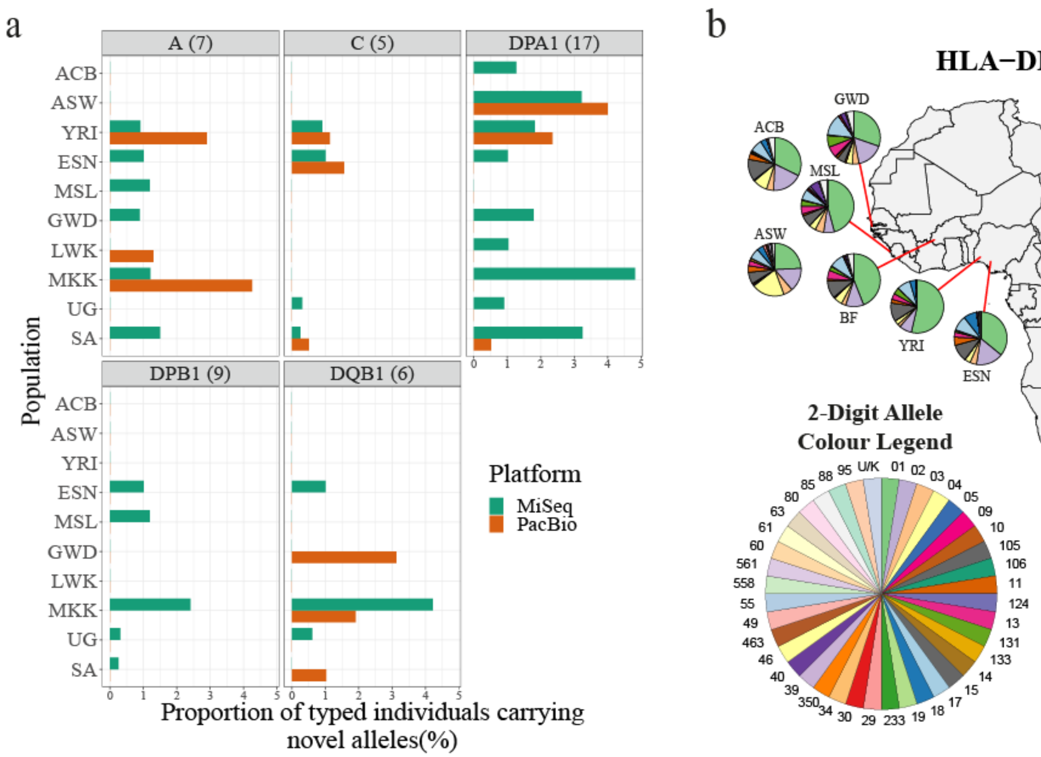

Supplementary Fig. 2.

Novel HLA alleles and diversity across Africa. (a) The proportion of individuals in each population with novel alleles confidently called using either MiSeq or PacBio calling pipelines. Total numbers of typed individuals can be found in **Supplementary Table 6**. **(b)** Pattern of differentiation of HLA-DPB1 2-digit alleles with frequencies plotted as pie-charts by population across Africa using a map available from the CIA World Data Bank II (2017 release).

ACB: African Caribbean in Barbados; ASW: African Ancestry in Southwest USA; BF: Burkina Faso; ESN: Esan in Nigeria; GWD: Gambian in Western Division, The Gambia – Mandinka; LWK: Luhya in Webuye, Kenya; MKK: Maasai in Kinyawa, Kenya; MSL: Mende in Sierra Leone; SA: South Africa; UG: Uganda; YRI: Yoruba in Ibadan, Nigeria.

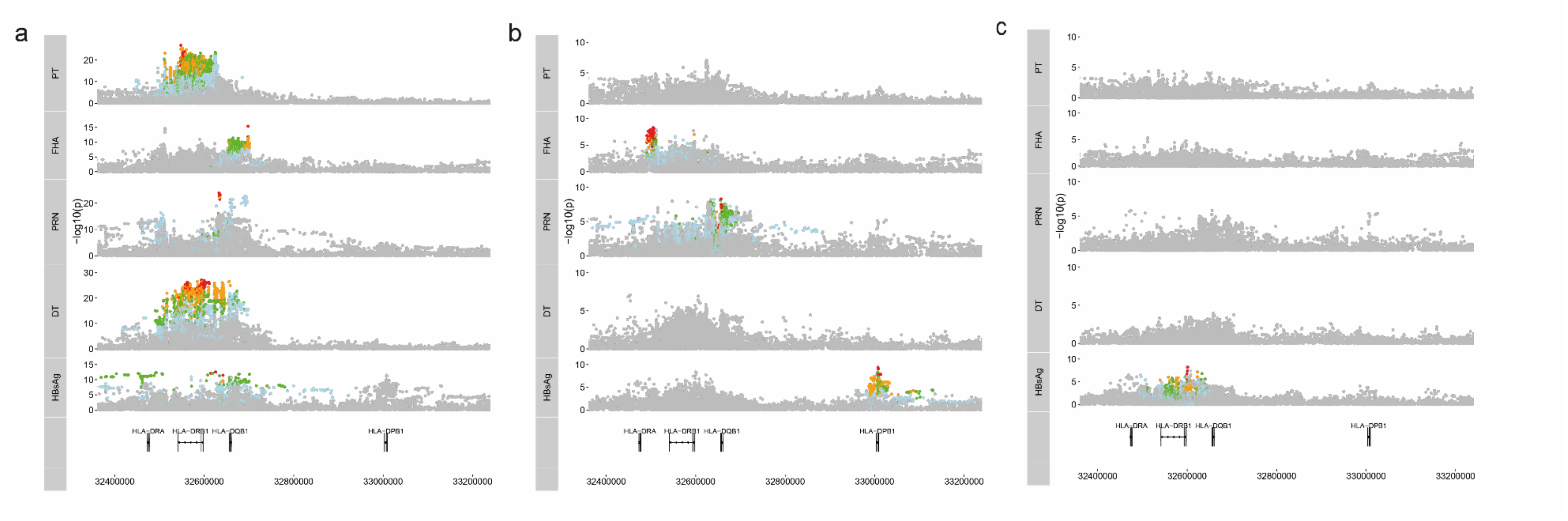

Supplementary Fig. 3.

Regional plots of association between SNP variants and antibody response traits with and without inclusion of top associated variants as covariates. (a) Unconditional analyses. **(b)** Analyses conditioning on the top associated variant from each individual pooled GWAS. **(c)** Analyses including both variants associated from unconditional and first round of conditional analyses for each individual pooled GWAS performed using linear mixed model regression**.** Wherever the index (most significant *P*-value) variant demonstrated significance $P<5x10^{-9}$, association peaks have SNPs colored by LD (r^2) with top associated variant (red 0.8-1; orange 0.6-0.8; green 0.4-0.6; blue 0.2-0.4), otherwise all points are colored in grey.

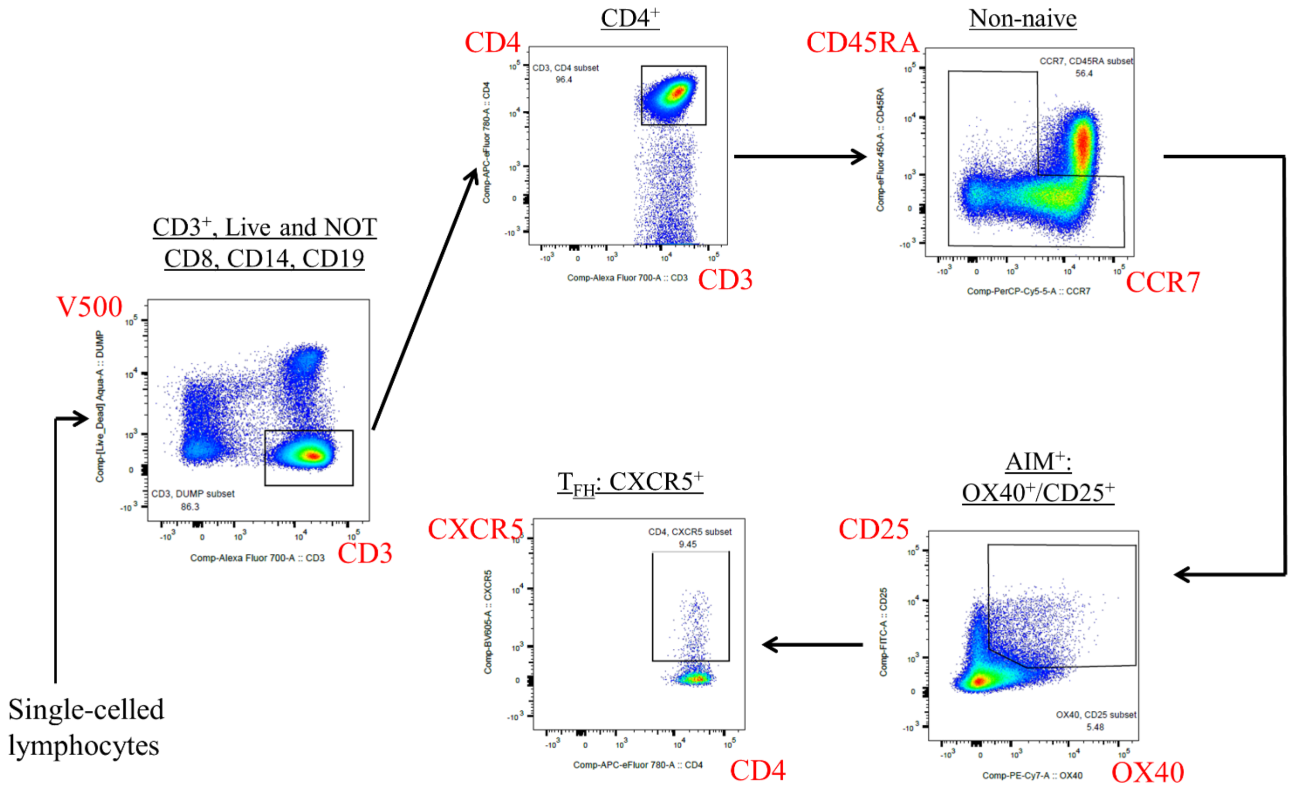

Supplementary Fig. 4.

Gating strategy to identify AIM non-naïve CD4+ T-cells and the T_{FH} (CXCR5+) subset.

Supplementary Table 1.

Descriptive characteristics of the individuals recruited into the study from three African *VaccGene* populations.

	Uganda	South Africa	Burkina Faso
Final genotyped number	1,391	755	353
Males, number (%)	706 (50.8)	384 (50.1)	177 (50.1)
HIV exposed, number (%)	130 (9.3)	431 (57.1)	NA
HIV infected, number (%)	19 (1.3)	1 (0.1)	NA
Age at serum/plasma sampling, mean (s.d.*)	54.1 (3.9)	26.4 (3.7)	87.4 (23.4)
Twin / triplet sets, number (%)	15 (1.1)	25 (3.3)	0 (0)
**Maternal Ethnicity**			
Baganda, number (%)	707 (50.8)		
Banyankole, number (%)	131 (9.4)		
Bunyarwanda, number (%)	76 (5.5)		
Busoga, number (%)	40 (2.8)		
Batooro, number (%)	65 (4.7)		
Luo, number (%)	78 (5.6)		
Other Ugandan, number (%)	294 (21.1)		
Zulu, number (%)		278 (36.8)	
Sotho, number (%)		186 (24.6)	
Xhosa, number (%)		112 (14.8)	
Tsonga, number (%)		54 (7.2)	
Tswana, number (%)		51 (6.8)	
Venda, number (%)		22 (1.6)	
Other South African, number (%)		72 (5.2)	
Gouin, number (%)			167 (47.3)
Karaboro, number (%)			65 (18.4)
Turka, number (%)			19 (5.4)
Peulh, number (%)			6 (1.7)
Other Burkinabe, number (%)			8 (2.3)
Unknown, number (%)	0	2 (0.3)	89 (25.2)

*: s.d.: standard deviation

NA: not available

Supplementary Table 2.

Summary of genotype quality control (QC) steps for each population. The number of individuals and variants removed within each population are presented.

		**Uganda**	**South Africa**	**Burkina Faso**
**Pre-QC**	Total number sent for genotyping	1,416	784	360
	Number of variants typed and mapping to Build 37	2,328,340	2,328,340	2,328,340
**Individual and autosomal variant QC**	Individuals failing call rate (<97%)	2	2	1
	Individuals with extreme heterozygosity (>3s.d.* around the mean)	13	13	5
	Individuals failing sex-check	2	2	1
	Individuals IBD>0.9 and not twins / triplets	8	12	0
	Autosomal variants with call rate <97%	73,540	66,464	79,400
	Autosomal variants in Hardy-Weinberg disequilibrium ($p<10^{-8}$)	27,002	19,629	11,647
**Chromosome X QC**	Chromosome X variants with call rate <97%	1,552	1,244	1,599
	Chromosome X variants in Hardy-Weinberg disequilibrium ($p<10^{-8}$)	45	29	8
**Post-QC**	Total individuals remaining	1,391	755	353
	Total autosomal variants remaining	2,176,695	2,191,144	2,186,190
	Total chromosome X variants remaining	45,725	47,292	46,868

*: s.d.: standard deviation

Supplementary Table 3.

Number of variants imputed into individual datasets divided between autosomes and the X-chromosomes. The total number of imputed variants includes all those listed in the output files direct from IMPUTE2. High quality SNPs are those only with an info score greater than 0.3 and those tested for association were only those of high quality with a minor allele frequency (MAF) greater than 0.01.

**Autosomal variants post-imputation**			
Cohort	Total imputed (n)	Total high quality (n)	Total tested for association (n)
Uganda	103,992,756	49,820,138	17,812,003
South Africa	103,948,407	39,414,539	20,351,192
Burkina Faso	103,543,613	31,941,883	18,537,816
**X-chromosome variants post-imputation**			
Uganda	4,257,294	1,792,278	724,497
South Africa	4,257,257	1,354,576	757,584
Burkina Faso	4,257,217	1,140,538	682,994

Supplementary Table 4 (Separate File).

Association statistics from the fixed-effect meta-analyses for all extended MHC variants (bi-allelic SNPs, HLA alleles and amino acids) in the three African populations for the five vaccine antibody responses with GWAS significant associations. The original results were derived from the pooled linear mixed model described in the main text. SNPs are coded in build 37 coordinates as 'chromosome':'base pair':'minor allele':'major allele'. Amino acids are coded as 'Gene'_'AA1'_'full length position'_'amino acid present'_'coding sequence position'. Coding sequence position was used in the main text. HLA alleles are coded as 'Gene'_'6 digit G Allele'.

Supplementary Table 5.

DNA samples for Maasai population.

ID	Sex	ID	Sex	ID	Sex	ID	Sex
NA21739	Male	NA21435	Male	NA21582	Male	NA21457	Female
NA21775	Female	NA21410	Male	NA21409	Female	NA21420	Male
NA21307	Male	NA21417	Male	NA21476	Female	NA21526	Female
NA21524	Female	NA21458	Male	NA21344	Male	NA21379	Female
NA21451	Female	NA21573	Male	NA21357	Female	NA21513	Female
NA21367	Male	NA21575	Male	NA21719	Male	NA21399	Male
NA21522	Male	NA21523	Male	NA21770	Female	NA21520	Male
NA21693	Female	NA21825	Female	NA21741	Male	NA21449	Female
NA21387	Male	NA21338	Male	NA21596	Male	NA21479	Female
NA21740	Male	NA21359	Male	NA21486	Female	NA21362	Female
NA21297	Female	NA21440	Male	NA21448	Male	NA21306	Female
NA21743	Male	NA21613	Female	NA21774	Female	NA21391	Female
NA21478	Male	NA21351	Male	NA21650	Female	NA21519	Male
NA21722	Male	NA21576	Female	NA21356	Male	NA21308	Female
NA21365	Female	NA21402	Male	NA21632	Female	NA21631	Male
NA21577	Male	NA21493	Female	NA21510	Female	NA21615	Female
NA21447	Male	NA21617	Female	NA21583	Male	NA21423	Male
NA21768	Female	NA21295	Male	NA21683	Female	NA21441	Female
NA21485	Male	NA21614	Male	NA21769	Female	NA21418	Female
NA21333	Female	NA21300	Female	NA21385	Female	NA21600	Female
NA21341	Male	NA21360	Female	NA21584	Male	NA21339	Female
NA21352	Male	NA21786	Female	NA21415	Female	NA21611	Female
NA21622	Female	NA21301	Male	NA21616	Male	NA21685	Male
NA21679	Female	NA21403	Female	NA21408	Male	NA21635	Female
NA21574	Female	NA21529	Female	NA21298	Male	NA21436	Female
NA21744	Male	NA21438	Female	NA21456	Male	NA21742	Male
NA21733	Female	NA21512	Male	NA21580	Female	NA21620	Female
NA21424	Female	NA21488	Male	NA21355	Male	NA21587	Male
NA21826	Female	NA21784	Female	NA21473	Female	NA21382	Female
NA21686	Female	NA21647	Male	NA21716	Male	NA21364	Female
NA21304	Male	NA21785	Female	NA21717	Female	NA21390	Male
NA21634	Male	NA21454	Female	NA21517	Female	NA21405	Male
NA21491	Female	NA21776	Female	NA21335	Male	NA21738	Male
NA21368	Female	NA21578	Female	NA21678	Male	NA21400	Female
NA21509	Male	NA21619	Male	NA21443	Male	NA21521	Male
NA21599	Male	NA21414	Male	NA21689	Male	NA21421	Female
NA21388	Female	NA21528	Male	NA21723	Female	NA21782	Female
NA21318	Male	NA21363	Female	NA21303	Female	NA21597	Female
NA21381	Male	NA21320	Female	NA21489	Female	NA21378	Male
NA21649	Male	NA21732	Male	NA21453	Male	NA21371	Female
NA21515	Male	NA21737	Male	NA21336	Female		
NA21316	Male	NA21353	Female	NA21450	Male		

Supplementary Table 6.

Number of individuals with intersecting genotype, MiSeq-based 6-digit 'G' resolution, PacBio (potentially 8-digit resolution), or Sanger-sequence based calls.

	Genotype*	MiSeq†	PacBio†								Sanger
			A	B	C	DRB1	DPB1	DQB1	DPA1	DQA1	
Uganda	330	330	0	0	0	0	0	0	0	0	47
Burkina Faso	167	167	0	0	0	0	0	0	0	0	0
South Africa	335	396	189	197	196	151	98	195	133	177	0
ACB	77	79	61	74	57	27	30	31	8	9	0
GWD	112	112	80	92	84	60	32	64	3	4	0
ESN	99	99	74	86	64	30	62	58	9	13	0
MSL	84	84	59	62	64	49	13	58	11	11	0
YRI	108	110	69	76	88	58	5	85	6	7	0
LWK	97	97	77	64	82	52	2	81	4	5	0
ASW	54	62	45	43	50	27	0	50	0	0	0
MKK	134	166	141	142	87	54	105	132	14	20	0
**Total**	**1597**	**1702**	**795**	**836**	**772**	**508**	**347**	**754**	**188**	**246**	**47**

*: Genotype data was either available from Omni2.5M genotype calling or next generation sequence data available intersecting with HLA type data of any type.

†: All PacBio data was available on individuals who also had MiSeq data, but not necessarily genotype data.

Supplementary Table 7.

Novel protein coding alleles discovered in the combined 1000Gp3-*VaccGene* dataset. Genbank accession numbers try to represent a single sequence for each novel allele submission.

**Gene**	**Reported Allele**	**Genbank Accession**	**WHO Reference**	**New Allele**
	02:XX	MH973915		NA*
	02:XX	MH973917		NA*
	02:XX	MH973918		NA*
HLA-A	23:XX	KU668724	10031574	23:73
	26:XX	KU668725	10031563	26:121
	30:XX	MK032383		NA
	32:XX	MG429694		32:106
HLA-B	15:XX	In submission		NA
	15:10:XX	MF170525		NA
	02:XX	In submission		NA
	02:10:XX	MH544312		02:10:04
HLA-C	04:XX	MH544322	10039321	04:368
	07:43P	KX017417	10032163	07:43:02
	07:XX	MG769797		07:629
	01:XX	In submission		NA*
	01:XX	In submission		NA*
	01:XX	MF170464		01:15
	02:XX	MF170461		NA*
	02:XX	MF170462		02:09
	02:XX	MF170456		NA*
	02:XX	MF170459		NA*
HLA-DPA1	02:XX	MH536331		02:12
	02:07:XX	NA		02:07:01*
	03:XX	In submission		NA*
	03:XX	MF170453		NA*
	03:01:XX	NA		03:01:02*
	03:02P	MF170463		NA
	04:XX	NA		04:02
	02:01:XX	MH974010		NA
	03:XX	In submission		NA
	11:XX	NA		654:01
HLA-DPB1	55:01:XX	MG805509		55:01:02
	104:01:XX	In submission		NA
	XX	KU668852	10031694	558:01
	XX	KU668853	10031698	561:01
	XX	NA		584:01
	01:XX	MK442249	10043295	NA
	01:XX	MF170427		NA
	01:01:XX	In submission		
HLA-DQA1	04:XX	MK442251		NA
	04:XX	MF170425		NA
	04:XX	MF170426		NA
	04:XX	In submission		NA
	02:XX	KU668797	10031657	02:70*
	02:XX			02:70*
HLA-DQB1	02:01P	MK058598		NA
	04:XX	MH536304		04:52
	04:XX	In submission		NA
	04:02:XX	NA		04:02:13

HLA-DRB1	03:XX	KU668802	10031673	03:131
	10:01:XX	MK192150	10042610	NA
HLA-DRB3	01:XX	MK058635		NA

*: These alleles may represent the same novel allele as others listed in the class but sequence reads are in the process of independent evaluation.

Supplementary Table 8 (Separate File).

Allele-specific statistics comparing imputed HLA allele calls from HLA*IMP:02 to sequence-based 6-digit ‘G’ typing divided by population. Calls are compared at 4-digit level of resolution and presented in 4-digit format. Locus A and allele 0101 refers to HLA-A*01:01 for example. New alleles defined through HLA typing are donated as XX and are detailed in **Supplementary Table 7**.

Supplementary Table 9 (Separate File).

Allele-specific statistics comparing imputed HLA allele calls from HLA*IMP:02G to sequence-based 6-digit 'G' typing divided by *VaccGene* population. Calls are compared at 4-digit level of resolution and presented in 4-digit format for comparison to **Supplementary Table 8**. Locus A and allele 0101 refers to HLA-A*01:01 for example. New alleles defined through HLA typing are donated as XX and are detailed in **Supplementary Table 7**.

Supplementary Table 10 (Separate File).

Allele-specific statistics comparing imputed HLA allele calls from HLA*IMP:02G to imputed HLA allele calls from the Broad Multi-Ethnic reference panel divided by *VaccGene* population. Calls are compared at 4-digit level of resolution and presented in 4-digit format for comparison to **Supplementary Table 9**. Locus A and allele 0101 refers to HLA-A*01:01 for example. New alleles defined through HLA typing are donated as XX and are detailed in **Supplementary Table 7**.

Supplementary Table 11.

Results from manual and automated step-wise modelling of class II HLA variants with five vaccine responses including principal components and time between sampling and vaccination as covariates. The reported SNP variants all had info scores greater than 0.8 (rs73727916 info 0.83-0.91 across the three cohorts; rs147857322 0.97-0.99 and rs34951355 0.88-0.91).

	**Method**	**Variant 1**[*]	**Variant 2**[*]	**Variant 3**[*]	**Variant 4**[*]	P_{LMM}	P_{uni}[†]	P_{multi}[‡]	**BIC**[§]
**PT**	Manual for HLA-DRB1 and HLA-DRB3	rs73727916	-	-	-	3.6x10^{-26}	8.1x10^{-30}	-	6442.55
		DRB3-74Gln↾	-	-	-	4.2x10^{-25}	2.0x10^{-28}	-	6453.00
		HLA-DRB3*02:02:01G↾	HLA-DRB3*03:01:01G↾	-	-	-	-	2.0x10^{-28}	6451.49
		DRB1-233Thr	-	-	-	-	1.7x10^{-26}	-	6457.75
	**Final**	**rs73727916**	**DRB3-74Gln**	-	-	-	-	3.3x10^{-35}	**6420.17**
**FHA**	Manual for HLA-DRB1	HLA-DRB1*08:04:01↾	-	-	-	4.8x10^{-15}	5.7x10^{-16}	-	6492.47
		DRB1-74Leu↾	-	-	-	4.9x10^{-15}	7.3x10^{-16}	-	6492.95
	Automated	HLA-DRB1*15:03:01G	-	-	-	2.6x10^{-8}	6.3x10^{-10}	-	6519.79
	**Final**	**HLA-DRB1*08:04:01**	HLA-DRB1*15:03:01G	-	-	-	-	1.8x10^{-21}	**6470.25**
**PRN**	Manual for HLA-DQB1	rs147857322	-	-	-	4.2x10^{-23}	1.1x10^{-25}	-	4469.76
		DQB1-74Ser↾	-	-	-	1.8x10^{-21}	4.6x10^{-25}	-	4472.52
		DQB1*05:01:01G↾	HLA-DQB1*04:02:01↾	-	-	-	-	3.7x10^{-27}	4463.84
	Automated	HLA-DRB1*11:02:01	-	-	-	-	2.3x10^{-13}	-	4525.74
		DQB1-175Glu	-	-	-	-	7.4x10^{-20}	-	4596.24
	**Final**	**rs147857322**	**DQB1-74Ser**	**HLA-DRB1*11:02:01**	**DQB1-175Glu**	-	-	1.4x10^{-38}	**4415.14**

***:** Variants associated with each trait identified through the pooled linear mixed model (pLMM) or univariate modelling approaches using linear regression.

†: The univariate model tested only on individuals restricted by IBD (< 0.2) using linear regression.

‡: Where multiple variants are tested in a conditional multivariable model the omnibus p-value is shown (p_{multi}).

§: The Bayesian Information Criterion (BIC) was calculated for all models. The most parsimonious model will have a BIC closer to 0. The model with the lowest BIC for each phenotype at each locus is shown in red and the BIC of the final model is shown in bold.

↾ For each locus and each trait any associated HLA amino acid was also tested in a univariate or multivariate model as described by classical HLA alleles that contain that residue. Although improved in the model when contained alone, DQB1-74Ser improved the final model when including the SNP variant in PRN.

Supplementary Table 11. continued

DT	Manual for HLA-DRB1	rs34951355	-	-	-	$1.5x10^{-26}$	$1.2x10^{-30}$	-	6347.03
	**Final**	**rs34951355**	-	-	-	$1.5x10^{-26}$	$1.2x10^{-30}$		**6347.03**
HBsAg	Manual for HLA-DRB1	DRB1-74Arg↑	-	-	-	$6.3x10^{-14}$	$1.9x10^{-18}$	-	5276.06
		HLA-DRB1*03:02:01	-	-	-	-	$6.3x10^{-15}$	-	5292.09
		HLA-DRB1*03↑	-	-	-	-	$1.9x10^{-18}$	-	5276.06
		HLA-DRB1*03:02:01↑	HLA-DRB1*03:01:01G↑	-	-	-	-	$3.1x10^{-18}$	5279.81
	Manual for HLA-DPB1	DPB1-85Gly↑	-	-	-	$1.2x10^{-10}$	$6.9x10^{-13}$	-	5301.34
		HLA-DPB1*105:01	-	-	-	-	$2.9x10^{-6}$	-	5331.00
		HLA-DPB1*02:01:02↑	HLA-DPB1*04:01:01G↑	HLA-DPB1*18:01↑	-	-	-	$4.3x10^{-9}$	5318.41
	Automated	DRB1-67Phe↑	-	-	-	-	$1.1x10^{-11}$	-	5306.81
		HLA-DRB1*08:04:01↑	HLA-DRB1*09:01:02G↑	HLA-DRB1*11:01:02↑	-	-	-	$1.8x10^{-11}$	5307.77
		DPB1-35Tyr	-	-	-	-	$1.7x10^{-13}$	-	5298.59
	**Final**	**DRB1-74Arg**	**DPB1-85Gly**	**DRB1-67Phe**	**DPB1-35Tyr**	-	-	$6.9x10^{-32}$	**5219.97**

§: The Bayesian Information Criterion (BIC) was calculated for all models. The most parsimonious model will have a BIC closer to 0. The model with the lowest BIC for each phenotype at each locus is shown in red and the BIC of the final model is shown in bold.

↑ For each locus and each trait any associated HLA amino acid was also tested in a univariate or multivariate model as described by classical HLA alleles that contain that residue.

Supplementary Table 12.

Signals of association between class II HLA variants and five vaccine response traits demonstrating significant evidence of heterogeneity for four out of the total 13 associated variants.

		Uganda			South Africa			Burkina Faso					
	**Variant**	**Beta**	**s.e.**	**MAF***	**Beta**	**s.e.**	**MAF***	**Beta**	**s.e.**	**MAF***	P_{FE}↑	P_{RE}‡	P_{Q}§
**PT**	DRB3-74Gln	-0.44	0.04	0.48	-0.04	0.05	0.40	-0.34	0.08	0.35	$3.1x10^{-28}$	0.05	$1.3x10^{-9}$
	rs73727916	0.43	0.04	0.40	0.16	0.06	0.37	0.24	0.08	0.52	$6.1x10^{-27}$	$3.1x10^{-3}$	$1.8x10^{-4}$
**FHA**	HLA-DRB1*15:03:01G	-0.24	0.06	0.15	-0.32	0.08	0.10	-0.33	0.11	0.16	$2.3x10^{-10}$	$2.3x10^{-10}$	NS
	HLA-DRB1*08:04:01	0.49	0.09	0.05	0.23	0.14	0.04	0.93	0.13	0.08	$1.2x10^{-16}$	$2.2x10^{-3}$	$1.1x10^{-3}$
**PRN**	HLA-DRB1*11:02:01	0.53	0.08	0.07	NA	NA	0.04	0.35	0.12	0.10	$3.8x10^{-16}$	$4.6x10^{-8}$	NS
	rs147857322	0.39	0.04	0.60	NA	NA	0.55	0.34	0.08	0.71	$2.0x10^{-26}$	$2.0x10^{-26}$	NS
	DQA1-175Glu	-0.25	0.05	0.22	NA	NA	0.30	-0.24	0.09	0.23	2.3x10-9	$2.3x10^{-9}$	NS
	DQB1-75Val	-0.32	0.04	0.45	NA	NA	0.43	-0.20	0.08	0.40	$6.1x10^{-17}$	$2.0x10^{-7}$	NS
**DT**	rs34951355	0.73	0.07	0.08	0.41	0.08	0.12	0.29	0.11	0.12	$1.2x10^{-29}$	$3.5x10^{-4}$	$3.9x10^{-4}$
**HBsAg**	DRB1-74Arg	-0.36	0.07	0.13	-0.36	0.07	0.18	-0.46	0.10	0.13	$4.6x10^{-16}$	$4.6x10^{-16}$	NS
	DRB1-67Phe	0.17	0.05	0.22	0.33	0.06	0.20	0.40	0.10	0.22	$6.4x10^{-12}$	$3.8x10^{-5}$	NS
	DPB1-35Tyr	-0.23	0.05	0.36	-0.25	0.05	0.43	-0.19	0.09	0.58	$3.2x10^{-12}$	$3.2x10^{-12}$	NS
	DPB1-85Gly	0.23	0.05	0.54	0.19	0.06	0.46	0.27	0.09	0.30	$4.2x10^{-11}$	$4.2x10^{-11}$	NS

* minor allele frequency

[↑] *P*value calculated from a fixed effects meta-analysis (P_{FE}) combining the effect estimates from the linear mixed model across the three studied populations.
[‡] *P*-value calculated from a random effects meta-analysis (P_{RE}) combining the effect estimates from the linear mixed model across the three studied populations.
[§] *P*-value of evidence of heterogeneity (P_Q) between the three studied populations calculated from the Cochran's Q statistic.

Supplementary Table 13 (Separate File).

Allele dosages and normalized antibody distributions for the 13 variants identified to be significantly associated with at least one antibody distribution from the imputation and fine-mapping exercise. Other relevant covariates including sex, genetic principal components 1-5 and time between last vaccine and sampling are all also provided. Data are available for the 2411 individuals with IBD<0.2, thus not requiring the genetic relatedness matrix or a mixed model to test for association.

Supplementary Table 14.

Impact of HIV exposure (*in utero*) or infection at birth, on antibody responses in Ugandan and South African infants. No data on HIV exposure was available in Burkina Faso. Pertactin was not administered to the South African infants as part of the acellular component of vaccine.

			PT (EU/ml)			FHA (EU/ml)			PRN (EU/ml)			DT (IU/ml)			HBsAg (mIU/ml)		
	**Status**	**Number**	**GMT[*]**	**CI[↑]**	**PVE[‡]**	**GMT[*]**	**CI[↑]**	**PVE[‡]**	**GMT[*]**	**CI[↑]**	**PVE[‡]**	**GMT[*]**	**CI[↑]**	**PVE[‡]**	**GMT[*]**	**CI[↑]**	**PVE[‡]**
**Uganda**	Unexposed and uninfected (U)	1242	14.8	13.7-16.1	-	4.4	4.1-4.6	-	13.2	12.4-13.9	-	34.2	32.1-36.5	-	103.3	94.1-113.3	-
	Exposed *in utero* but not infected at birth (E)	130	18.54	14.6-23.6	-	4.4	3.7-5.3	-	13.5	11.2-16.1	-	35.8	29.8-43.0	-	122.5	93.7-160.3	-
	Infected at birth (I)	19	4.1	2.8-6.0	1.0	3.6	2.6-4.8	0.0	4.5	3.0-6.7	1.4	6.7	4.4-10.3	2.7	19.7	11.5-33.8	1.7
**South Africa**	Unexposed and uninfected (U)	335	75.4	69.3-82.1	-	109.7	100.3-120.0	-	NA	NA	-	339.2	310.0-371.1	-	603.6	556.1-655.2	-
	Exposed *in utero* but not infected at birth (E)	419	75.4	70.7-80.4	-	100.4	92.7-108.8	-	NA	NA	-	319.6	295.7-345.4	-	660.0	616.1-707.0	-
	Infected at birth (I)	1	79.7	NA	NA	141.1	NA	NA	NA	NA	NA	277.2	NA	NA	1001	NA	NA

[*] geometric mean titre (GMT)
[↑] 95% confidence interval (CI) around the geometric mean
[‡] proportion of variance (PVE) explained by HIV status defining unexposed and exposed compared against infected infants

Supplementary Table 15.

Characteristics of donors and PBMC samples used for PT-specific T_{FH} assay.

	**DRB1-233Arg (Number (%))**	**DRB1-233Thr (Number (%))**
Final number	15	14
Males	7 (46.7)	6 (42.9)
Mean age at sampling in years	46.1 (23.7)†	43.2 (24.3) †
Mean storage time of PBMCs in years	3.87 (2.1) †	3.81 (2.7) †
**Participant Ethnicity**		
Asian	0	1 (7.1)
Pacific Islander	1 (6.7)	3 (21.4)
Black	6 (40.0)	0
Hispanic / Latino	3 (20.0)	0
White	4 (26.7)	9 (64.2)
Unknown	1 (6.7)	1 (7.1)
**4-Digit HLA-DRB1 Alleles**		
01:01	0	9 (32.1)
01:02	0	2 (7.1)
03:01	4 (13.3)	0
03:02	1 (3.3)	0
03:17	1 (3.3)	0
11:01	7 (23.3)	0
11:02	4 (13.3)	0
11:04	4 (13.3)	0
13:01	4 (13.3)	0
13:02	1 (3.3)	0
13:03	2 (6.7)	0
13:04	2 (6.7)	0
15:01	0	9 (32.1)
15:02	0	7 (25.0)
15:09	0	1 (3.6)

†: values given in mean (standard deviation)

Supplementary Table 16.

Breadth of PT-peptides binding to associated HLA-DRB1 alleles compared to the breadth of peptides derived from TT binding to the same alleles.

	Allele	PT Breadth†	TT Breadth†	*P*-value
**DRB1-233Thr**	DRB1*01:01	3.1	4.6	
	DRB1*01:02	24.2	26.3	
	DRB1*15:01	3.5	11.3	
	DRB1*15:03	3.1	8.1	
	Average	8.5 (+/- 10.5)	12.6 (+/- 9.5)	NS
**DRB1-233Arg**	DRB1*03:01	22.03	26.7	
	DRB1*11:01	9.3	6.5	
	DRB1*11:02	0.4	7.5	
	DRB1*13:02	12.3	22.4	
	Average	11.0 (+/- 8.9)	15.8 (+/- 10.3)	NS

†: Values represent % of peptides derived from the corresponding toxin that are predicted to bind with high affinity (+/- standard deviation) calculated according to the 5th percentile rank in IEDB. Significance tested using 2-tailed Mann-Whitney test. NS: not significant.

Supplementary Table 17 (Separate File).

Summary beta, standard error and *P*-values for fixed effects meta-analysis of *cis*-QTL analyses for each of eight major class I and II *HLA* genes. The original statistics were calculated using linear regression of the derived gene expression level in each individual population.

Supplementary Table 18.

Breadth of DT-peptides binding to either HLA-DRB1 alleles carried on HLA-DRB4 or non-HLA-DRB4 haplotypes, in comparison to DRB4*01 alleles, when compared to the breadth of peptides derived from TT binding to the same alleles.

	**Allele**	**DT Breadth**†	**TT Breadth**†	***P*-value**
**Non DRB4-linked DRB1 alleles**	DRB1*01:01	2.3	4.6	
	DRB1*01:02	18.3	26.3	
	DRB1*03:01	15.6	20.7	
	DRB1*03:02	2.0	6.7	
	DRB1*08:04	19.8	31.3	
	DRB1*10:01	1.5	7.3	
	DRB1*11:01	13.2	22.8	
	DRB1*11:02	23.4	26.7	
	DRB1*12:01	3.1	5.5	
	DRB1*13:01	18.5	29.7	
	DRB1*13:02	7.1	7.6	
	DRB1*13:03	2.2	6.1	
	DRB1*15:03	3.5	11.3	
	Average	10.0 (+/- 8.2)	15.9 (+/- 10.4)	NS
**DRB4-linked DRB1 alleles**	DRB1*04:01	8.2	18.4	
	DRB1*04:05	5.5	17.6	
	DRB1*07:01	9.3	7.7	
	DRB1*09:01	9.9	9.8	
	Average	8.2 (+/- 1.9)	13.4 (+/- 5.4)	NS
**HLA-DRB4**	DRB4*01:01	4.0	6.1	
	DRB4*03:01	3.6	3.4	
	Average	3.8 (+/- 0.28)	4.75 (+/- 1.9)	NS

†: Values represent % of peptides derived from the corresponding toxin that are predicted to bind with high affinity (+/- standard deviation) calculated according to the 5th percentile rank in IEDB. Significance tested using 2-tailed Mann-Whitney test. NS: not significant.
