# Supplementary material for: High-resolution African HLA resource uncovers HLA-DRB1 expression effects underlying vaccine response
Source: Nat Med. 2024 May 13;30(5):1384–94. doi: 10.1038/s41591-024-02944-5 (PMC11108778; doi:10.1038/s41591-024-02944-5)
Supplement: Supplementary file 2 — Reporting Summary [file 41591_2024_2944_MOESM2_ESM.pdf]

nature portfolio

Corresponding author(s): Alexander J Mentzer

Last updated by author(s): Mar 1, 2024

Reporting Summary

Nature Portfolio wishes to improve the reproducibility of the work that we publish. This form provides structure for consistency and transparency in reporting. For further information on Nature Portfolio policies, see our Editorial Policies and the Editorial Policy Checklist.

Statistics

For all statistical analyses, confirm that the following items are present in the figure legend, table legend, main text, or Methods section.

n/a	Confirmed	
☐	☒	The exact sample size (*n*) for each experimental group/condition, given as a discrete number and unit of measurement
☐	☒	A statement on whether measurements were taken from distinct samples or whether the same sample was measured repeatedly
☐	☒	The statistical test(s) used AND whether they are one- or two-sided<br>*Only common tests should be described solely by name; describe more complex techniques in the Methods section.*
☐	☒	A description of all covariates tested
☐	☒	A description of any assumptions or corrections, such as tests of normality and adjustment for multiple comparisons
☐	☒	A full description of the statistical parameters including central tendency (e.g. means) or other basic estimates (e.g. regression coefficient) AND variation (e.g. standard deviation) or associated estimates of uncertainty (e.g. confidence intervals)
☐	☒	For null hypothesis testing, the test statistic (e.g. *F*, *t*, *r*) with confidence intervals, effect sizes, degrees of freedom and *P* value noted<br>*Give P values as exact values whenever suitable.*
☒	☐	For Bayesian analysis, information on the choice of priors and Markov chain Monte Carlo settings
☒	☐	For hierarchical and complex designs, identification of the appropriate level for tests and full reporting of outcomes
☐	☒	Estimates of effect sizes (e.g. Cohen's *d*, Pearson's *r*), indicating how they were calculated

Our web collection on statistics for biologists contains articles on many of the points above.

Software and code

Policy information about availability of computer code

Data collection	VaccGene data collection:<br>- Microsoft Excel 2016 (16.0.5435.100)<br>ALSPAC<br>- variable search data tool http://www.bristol.ac.uk/alspac/researchers/our-data/<br>HLA typing<br>- HistoS and HistoTyper software (commercial)<br>- uTYPE (version 7)<br>VaccGene genotyping:<br>- HumanOmni 2.5M-8 ('octo') BeadChip array version 1.1<br>- Illuminus (commercial)<br>- GenCall (commercial)<br>MKK sequencing<br>- Illumina sequencing control software (SCS version 3.3.76)<br>- Wellcome Sanger Institute BAMBI software (version 0.9.4)<br>- BWA MEM (version 0.7.15-r1140)<br>- scramble (version 1.14.8)<br>- samtools reheader (version 1.3.1-npg-Sep2016)<br>- biobambam (version 2.0.65)<br>- GATK HaplotypeCaller (version 3.8-0-ge9d806836)<br>- GATK CombineGVCFs (version 2017-11-07-g45c474f)<br>- LiftOver (https://genome.sph.umich.edu/wiki/LiftOver)

Data analysis

Genotyping:
- PLINK (v1.7)
- EIGENSOFT (v4.2)
- SHAPEIT2 (v2.r790)
- IMPUTE2 (v2.3.2)
- ADMIXTURE (v1.3.0)
Population genetics:
- 'diveRsity' package in R v3.5.1
- ADMIXTURE (v1.3.0)
Antibody response analysis
- R v3.5.1
Association testing:
- GEMMA (v0.94)
- METASOFT (v1.0)
HLA imputation
- HLA IMP:02 March 2016 reference panel release
- Broad multi-ethnic HLA imputation reference panel version 1.0
RNA sequencing
- FastQC v0.11.8
- STAR 2.6.0c
Flow cytometry
- Flowjo 10.3

For manuscripts utilizing custom algorithms or software that are central to the research but not yet described in published literature, software must be made available to editors and reviewers. We strongly encourage code deposition in a community repository (e.g. GitHub). See the Nature Portfolio guidelines for submitting code & software for further information.

Data

Policy information about availability of data

All manuscripts must include a data availability statement. This statement should provide the following information, where applicable:

- Accession codes, unique identifiers, or web links for publicly available datasets
- A description of any restrictions on data availability
- For clinical datasets or third party data, please ensure that the statement adheres to our policy

All direct genotypes from VaccGene individuals post-quality control alongside imputed data and raw and curated HLA sequence data and calls have been submitted to the European Genome-Phenome Archive under accession EGAS00001000918, with the datasets under EGAD00010002578 and EGAD00010002583 (Uganda); EGAD00010002582 and EGAD00010002580 (South Africa); EGAD00010002581 and EGAD00010002579 (Burkina Faso). The merged SNV calls for the African populations and the HLA allele calls and related sequence data are found under EGAD00010002577 and EGAD00001011379 respectively. Data is available to researchers following application to the Wellcome Sanger Institute Data Sharing team (datasharing@sanger.ac.uk with details available at https://edam.sanger.ac.uk/) and review of an application by a Data Access Committee. The Committee are committed to rapid decision making and ready access to data and will endeavor to make a decision on any requests received within 2 weeks of receipt to the Committee. Summary statistics for the genome-wide association tests of imputed data for eight vaccine antibody levels are available on Zenodo (https://doi.org/10.5281/zenodo.7357687). The RNA sequencing data for 1000 Geneomes is available at ENCODE https://www.encodeproject.org/search/?searchTerm=AFGR&type=Experiment, and data for DICE is available from https://dice-database.org/downloads. HLA-peptide binding data was derived from the Immune Epitope Database (IEDB) accessed in 2017 and 2018, and the antigen sequences were downloaded from SWISSPROT.

Research involving human participants, their data, or biological material

Policy information about studies with human participants or human data. See also policy information about sex, gender (identity/presentation), and sexual orientation and race, ethnicity and racism.

Reporting on sex and gender

Sex of potential or consenting participants was not considered specifically relevant to the design or performance of this study including in the components where cell lines or stored samples were used. The only aspects where sex was important was to approach and recruit mothers during pregnancy for the recruitment of the children who would have data analysed as part of the VaccGene component of this study, and to allow a 'sex-check' comparing reported and genetically determined sex as a standard component of genotype quality control to minimise risks of including samples that may have been subject to a mix-up. No consideration was taken in terms of inclusion of the children based on sex. All analyses and presentations of data are based on self-reported or genetically determined sex, where applicable, and these were only performed as secondary analyses. The breakdown of VaccGene participants by self-reported sex are provided in Table S1. Consent is provided to share individual-level sex data which are available as part of the data availability for VaccGene as detailed above.

Reporting on race, ethnicity, or other socially relevant groupings

The children who were included in the VaccGene study had their ethnicity defined according to their mother's self-reported ethnicity. These definitions were decided by the consenting guardians of the participants themselves and the breakdown is provided in Table S1, stratified by country of recruitment. Other samples included in the analyses from 1000 Genomes were classified according to the original classifications used as part of the 1000 Genomes Project. The independent sample-set used for flow analysis collected information of participants' self-reported ethnicity, provided in Table S10.

Population characteristics

All data relates to either genetic data or context-specific RNA expression levels and therefore age was not considered relevant in this study. The populations of individuals included in this study are clearly indicated in the manuscript and were chosen to be representative of diverse African populations through either targeted recruitment in this study (VaccGene including individuals from Uganda, Burkina Faso and South Africa), or from established population studies including 1000

Genomes. A breakdown of the self-assigned ancestries are provided for the three VaccGene populations in Table S1.

Recruitment

Dedicated recruitment for this study was dependent upon the VaccGene study. Participants included in the VaccGene study were recruited from three African countries selected partly due to their geographic dispersal across the continent and partly due the availability of high quality metadata and biological samples relevant to infant vaccination. These sites were in Uganda, South Africa and Burkina Faso. Individuals from each of the cohorts were included if their dates of birth, vaccination and blood sampling were available and if it was confirmed that they had received three doses of vaccines including diphtheria toxin (DT), tetanus toxin (TT), pertussis antigens, Haemophilus influenzae (Hib), and hepatitis B surface antigen (HBsAg) and a single dose of measles virus (MV) vaccine. The receipt of vaccines was confirmed through referencing the vaccination cards of infant participants or documented administration of vaccines by the research teams where relevant. Beyond exclusion criteria involved in preliminary recruitment of the individuals, no further exclusion occurred based on gender, ethnicity, HIV exposure or any other health status.

Uganda: The Entebbe Mother and Baby Study (EMaBS) is a prospective birth cohort that was originally designed as a randomised controlled trial to test whether anthelminthic treatment during pregnancy and early infancy was associated with differential response to vaccination or incidence of infections such as pneumonia, diarrhea or malaria (http://emabs.lshtm.ac.uk/). EMaBS originally recruited 2,507 women between 2003 and 2006; 2,345 livebirths were documented and 2,115 children were still enrolled at 1 year of age. Pregnant women in the second or third trimester were enrolled at Entebbe Hospital antenatal clinic if they were resident in the study area, planning to deliver in the hospital, willing to know their HIV status and willing to take part in the study. They were excluded if they had evidence of possible helminth-induced pathology (severe anemia, clinically apparent liver disease, bloody diarrhea), if the pregnancy was abnormal, or if they had already enrolled during a previous pregnancy. The mothers and infants underwent intensive surveillance during the first year of infant life. Blood samples were taken and stored from both mother and cord blood around the time of birth. Samples, including whole blood, were then obtained from the child annually. The primary results of the clinical trial demonstrated that anthelminthic treatment during pregnancy had no effect on infant response to BCG, tetanus, or measles immunisation, or the risk of subsequent infectious diseases. All infants under follow up had a sample of whole blood collected annually on or around their birthday (2-5 ml depending on the age). The child's samples were subsequently divided into plasma and red cell pellets as described in more detail below. Infants were included in the present study if 1) receipt of three doses of DTwP/Hib/HBV (at approximately 6, 10 and 14 weeks of age) and one dose of MV vaccine (at 9 months of age) could be confirmed as being administered by the research team or from their vaccination records 2) DNA could be extracted from stored red cell pellets 3) plasma samples were available from the 12 month age point of sampling. Informed written consent was re-acquired from the mothers or guardians, and where appropriate consent from the child and assent from the guardian or mother, specifically for the genetic component of this study.

South Africa: The Soweto Vaccine Response Study included six-month old infants born in Chris Hani Baragwanath Hospital living in the Soweto region of Johannesburg, South Africa and who were identified from screening logs and databases of participants involved in vaccine clinical trials coordinated by the Vaccine and Infectious Diseases Analytics (Wits-VIDA) Unit (https://wits-vida.org). Mothers had originally participated in a randomised, double-blind, placebo-controlled clinical trial in 2011 and 2012 on the safety, immunogenicity, and efficacy of trivalent inactivated influenza vaccine during pregnancy, where the trials had demonstrated that the vaccine was immunogenic and provided partial protection against influenza. Mothers of the infants were approached if the infants had received all of their vaccines up to six months of age (DTaP/Hib/HBV at approximately 4, 8 and 12 weeks of age). The infants were sampled prospectively at six months of age and at 12 months after receipt of MV vaccine at 9 months. Single whole blood samples were collected and prepared using a similar protocol to that used in Entebbe to extract DNA from cell pellets and plasma for antibody assays.

Burkina Faso: The VAC050 ME-TRAP Malaria Vaccine Trial included infants between the ages of 6 and 18 months living in the Banfora region of Burkina Faso who were recruited into a Phase 1/2b clinical trial to test the safety, immunogenicity and efficacy of an experimental heterologous viral-vectored prime-boost liver-stage malaria vaccine. These infants were all expected to receive their EPI vaccines (DTwP/Hib/HBV) as part of the usual national schedule at 4, 8 and 12 weeks of age. Infants were precluded from participating in the trial if they were found to have clinical or hematological (venous hemoglobin less than 8 g/dL) evidence of severe anemia, history of allergic or neurological disease or malnutrition. The primary endpoint of the trial has been published demonstrating that the vaccine is safe and immunogenic but had no protective efficacy against clinical malaria. Out of a total of 730 infants that were recruited into the study following informed and written consent from the mother, samples suitable for extraction of DNA were collected and stored from 400 infants (350 vaccine recipients and 50 recipients of a control rabies vaccine). Samples of plasma were available from the infants at multiple time-points following the experimental vaccine receipt. Samples from individuals taken at time points as close to the 12-month age as possible were prioritised for EPI vaccine response measurements. The infants underwent intensive clinical history and examination during screening and follow-up.

Ethics oversight

Ethical approval for the Ugandan study was provided locally by the Uganda Virus Research Institute (reference GC/127/12/07/32) and Uganda National Council for Science and Technology (MV625), and in the UK by London School of Hygiene and Tropical Medicine (A340) and Oxford Tropical Research (39-12 and 42-14) Ethics Committees.

In South Africa the mothers were consented after receiving information about the study in accordance with ethical approval from the University of Witwatersrand Human Research Ethics Committee (reference M130714) and the Oxford Tropical Research Ethics Committee (1042-13 and 42-14).

In Burkina Faso, the mothers of the participating infants provided consent for their children to be enrolled in the clinical trial and for subsequent genetic studies to be undertaken for all vaccines received in accordance with ethical approval from the Ministere de la Recherche Scientifique et de l'Innovation in Burkina Faso (reference 2014-12-151) and the Oxford Tropical Research Ethics Committee (41-12).

Note that full information on the approval of the study protocol must also be provided in the manuscript.

Field-specific reporting

Please select the one below that is the best fit for your research. If you are not sure, read the appropriate sections before making your selection.

☒ Life sciences ☐ Behavioural & social sciences ☐ Ecological, evolutionary & environmental sciences

For a reference copy of the document with all sections, see nature.com/documents/nr-reporting-summary-flat.pdf

Life sciences study design

All studies must disclose on these points even when the disclosure is negative.

Sample size	For the vaccine antibody discovery, we estimated that a sample size of 2,500 individuals would have 94.7% power to identify variants explaining 2% of the variance of antibody responses with a P-value threshold of observing an association due to chance (α) of 1 x 10-8 using the Genetic Power Calculator (http://zzz.bwh.harvard.edu/gpc/).
Data exclusions	Genetic samples were removed if they had high genotyping missingness, genetic sex did not match reported sex or they had high heterozygosity — these are metrics of poor sample or genotyping quality. Individuals were excluded if they had estimates ≥0.9 identity by descent, excluding the individual with the highest SNP missingness rate from each pair preferentially — to remove duplicated samples.
Replication	Three independent studies were included within VaccGene to enable cross-comparison between populations and internal replication. The signals of association at HLA were replicated in all three cohorts as shown in Fig. 3.
Randomization	No randomisation was used in the evaluation of the primary results reported in our analyses. No randomisation was applied during the recruitment or follow up of South African infants. Infants in the Ugandan study had been randomised to anthelminthic treatment and Burkinabe participants had been randomised to a malaria vaccine candidate, but neither of these interventions were found to exert and effect on measured vaccine responses and so were not analysed further. Other covariates influencing vaccine responses were explored as shown in Fig. 4 and Extended Data Fig. 7. Given the small impact of other factors, only the time between last vaccine and sampling was used as a covariate in analyses.
Blinding	The phenotypes here were all quantitative following known vaccination and so blinding was not applicable.

Reporting for specific materials, systems and methods

We require information from authors about some types of materials, experimental systems and methods used in many studies. Here, indicate whether each material, system or method listed is relevant to your study. If you are not sure if a list item applies to your research, read the appropriate section before selecting a response.

Materials & experimental systems

n/a	Involved in the study	
☐	☒	Antibodies
☒	☐	Eukaryotic cell lines
☒	☐	Palaeontology and archaeology
☒	☐	Animals and other organisms
☐	☒	Clinical data
☒	☐	Dual use research of concern
☒	☐	Plants

Methods

n/a	Involved in the study	
☒	☐	ChIP-seq
☐	☒	Flow cytometry
☒	☐	MRI-based neuroimaging

Antibodies

Antibodies used

The antibody panel used in the flow experiment included antibodies as follows: CCR7-PerCP-Cy5.5 (G043H7, catalogue 353220), Biolegend, San Diego, USA:
OX40-PE-Cy7 (BerACT35, catalogue 350012)
CXCR5-Brilliant Violet 605 (J252D4, catalogue 356930)

eBioscience, San Diego, USA
CD45RA-eFluor450 (HI100, catalogue 48-0458-42)
CD4-APC-eFluor780 (RPA-T4, catalogue 47-0049-42)

BD Biosciences, San Jose, USA
CD25-FITC (M-A251, catalogue 560990)
CD14-V500 (M5E2, catalogue 561392)
CD19-V500 (HIB19, catalogue 561125)
CD8-V500 (RPA-T8, catalogue 560775)

Thermo-Fisher Scientific, Waltham, USA

LIVE/DEAD Aqua stain (catalogue L34957)

Jackson ImmunoResearch Laboratories Inc. (Westgrove, Pa) for the Luminex assay
Anti-human IgG antibody conjugated to R-phycoerythrin (R-PE), catalogue 109-116-170

Validation

OX40-PE-Cy7 (from product sheet): PHA-stimulated (3 days) human peripheral blood lymphocytes were stained with CD4 FITC and OX-40 (clone Ber-ACT35) PE/Cyanine7 or mouse IgG1, κ PE/Cyanine7 isotype control and the former demonstrated higher fluorescence intensity.

CXCR5-Brilliant Violet 605 (from product sheet):Human peripheral blood lymphocytes were stained with CD19 APC and CD185 (clone J252D4) Brilliant Violet 605™ or mouse IgG1, κ Brilliant Violet 605™ isotype control and the former demonstrated higher fluorescence intensity.

CD45RA-eFluor450 (from website): Staining of normal human peripheral blood cells with Anti-Human CD45RO FITC (Product # 11-0457-42) and Mouse IgG2b K Isotype Control eFluor® 450 (Product # 48-4732-82) or Anti-Human CD45RA eFluor® 450 with the latter demonstrating higher fluorescence intensity.

CCD4-APC-eFluor780 (from website): Staining of normal human peripheral blood cells with Anti-Human CD8a APC (Product # 17-0088-42) and Mouse IgG1 kappa Isotype Control APC-eFluor® 780 (Product # 47-4714-82) or Anti-Human CD4 APC-eFluor® 780 with the latter demonstrating higher fluorescence intensity.

CD25-FITC (from website): Flow cytometric analysis of CD25 expression on PHA-stimulated human peripheral blood lymphocytes. Phytohemagglutinin-stimulated (3 days) PBMCs were stained with either FITC Mouse Anti-Human CD25 (Cat. No. 555431/560990) or FITC Mouse IgG1 κ Isotype Control (Cat. No. 555748), with the human demonstrating greater fluorescence intensity.

CD14-V500 (from website): Flow cytometric analysis of CD14 expressed on human peripheral blood monocytes. Human whole blood was stained with the BD Horizon™ V500 Mouse Anti-Human CD14 antibody (Cat. No. 561391/561392) or with a BD Horizon™ V500 Mouse IgG2a, κ isotype control (Cat. No. 561221). The erythrocytes were lysed with BD Pharm Lyse™ Lysing Buffer (Cat. No. 555899). The fluorescence histograms were derived from events with the forward and side light-scatter characteristics of viable monocytes. Flow cytometry was performed using a BD™ LSR II Flow Cytometer System and the anti-human antibody showed higher fluorescence intensity.

CD8-V500 (from website): Flow cytometric analysis of CD8 on human lymphocytes. Whole blood was stained with BD Horizon™ V500 Mouse Anti-Human CD8 (Cat. No. 560775/560774;) and compared to whole blood stained with BD Horizon™ V500 Mouse IgG1, κ Isotype Control (Cat. No. 560787). Lymphocytes were selected by light scatter profile. Flow cytometry was performed on a BD FACSCanto™ II flow cytometry system and the anti-human antibody showed higher fluorescence intensity.

CD19-V500 (from website): Flow cytometric analysis of CD19 expression on human peripheral blood lymphocytes. Whole blood was stained with BD Horizon™ V500 Mouse anti-Human CD19 antibody (Cat. No. 561121) or with a BD Horizon™ V500 Mouse IgG1, κ Isotype Control (Cat. No. 560787). The erythrocytes were lysed with BD PharmLyse™ Lysing Buffer (Cat. No. 555899). The fluorescence histograms were derived from events with the forward and side light-scatter characteristics of viable lymphocytes. Flow cytometry was performed using a BD FACSCanto™ II Flow Cytometer System and the anti-human antibody showed higher fluorescence intensity.

LIVE/DEAD Aqua stain: the validation is presented here: https://www.thermofisher.com/document-connect/document-connect.html?url=https://assets.thermofisher.com/TFS-Assets%2FCMD%2Fposters%2F2009-dead.pdf

Anti-human IgG antibody conjugated to R-PE (from website): "Based on immunoelectrophoresis and/or ELISA, the antibody reacts with the Fc portion of human IgG heavy chain but not with the Fab portion of human IgG. No antibody was detected against human IgM or IgA, or against non-immunoglobulin serum proteins. The antibody has been tested by ELISA and/or solid-phase adsorbed to ensure minimal cross-reaction with bovine, mouse and rabbit serum proteins, but it may cross-react with immunoglobulins from other species."

Clinical data

Policy information about clinical studies
All manuscripts should comply with the ICMJE guidelines for publication of clinical research and a completed CONSORT checklist must be included with all submissions.

Clinical trial registration

Entebbe Mother and Baby Study is a unique birth cohort, on-going in Africa. The study was designed as a trial [ISRCTN32849447], to investigate possible benefits of treating worm infections during pregnancy and early childhood.
The Soweto Vaccine Response Study included six-month old infants born in Chris Hani Baragwanath Hospital living in the Soweto region of Johannesburg, South Africa who were identified from screening logs and databases of participants involved in vaccine clinical trials coordinated by the Vaccine and Infectious Diseases Analytics (Wits-VIDA) Unit (https://wits-vida.org). These studies are described in DOI: 10.1056/NEJMoa1401480 with available protocols.
The VAC050 ME-TRAP Malaria Vaccine Trial (NCT01635647) recruited infants between the ages of 6 and 18 months living in the Banfora region of Burkina Faso who were recruited into a Phase 1/2b clinical trial to test the safety, immunogenicity and efficacy of an experimental heterologous viral-vectored prime-boost liver-stage malaria vaccine.

Study protocol

http://emabs.lshtm.ac.uk/
DOI: 10.1056/NEJMoa1401480
https://clinicaltrials.gov/ct2/show/NCT01635647?term=NCT01635647&draw=2&rank=1

Data collection

The samples used for antibody assays for the Ugandan study were collected from the participants when they reached one year of age between 2003 and 2006. DNA was extracted from stored samples collected on a birthday when the child was anywhere between one

and five years of age. Vaccine and demographic data were collected by trial staff local to Entebbe during the first year of child life.

The samples for the South African antibody and DNA analysis were collected form the infants at 6 months of age between the years 2013-2014. Demographic and vaccine data was collected by trial staff local to South Africa during the first year of child life.

The samples for the Burkinabe vaccine antibody and genetic analysis were collected from the participants at between 6 and 18 months of age between the years of 2012-2014. Data on vaccine receipt and demographics were collected throughout the same time-period of child participation in the vaccine trial by trial staff based locally in Banfora.

Outcomes

The primary outcomes of the study detailed here included the measurement of antibody reponses against eight vaccine antigens, and the characterisation of genome-wde variation in the participating infants at between 6 and 18 months of age. The antibody levels were measured using either a Luminex multiplex assay (for pertussis, tetanus, diphtheria, Haemophilus and measles antigens), or a dedicated ELISA platform for hepatitis B. The genetic data was derived using genome wide genotyping undertaken on an Illumina 2.5M array.

Plants

Seed stocks

Report on the source of all seed stocks or other plant material used. If applicable, state the seed stock centre and catalogue number. If plant specimens were collected from the field, describe the collection location, date and sampling procedures.

Novel plant genotypes

Describe the methods by which all novel plant genotypes were produced. This includes those generated by transgenic approaches, gene editing, chemical/radiation-based mutagenesis and hybridization. For transgenic lines, describe the transformation method, the number of independent lines analyzed and the generation upon which experiments were performed. For gene-edited lines, describe the editor used, the endogenous sequence targeted for editing, the targeting guide RNA sequence (if applicable) and how the editor was applied.

Authentication

Describe any authentication procedures for each seed stock used or novel genotype generated. Describe any experiments used to assess the effect of a mutation and, where applicable, how potential secondary effects (e.g. second site T-DNA insertions, mosiacism, off-target gene editing) were examined.

Flow Cytometry

Plots

Confirm that:

☒ The axis labels state the marker and fluorochrome used (e.g. CD4-FITC).

☒ The axis scales are clearly visible. Include numbers along axes only for bottom left plot of group (a 'group' is an analysis of identical markers).

☒ All plots are contour plots with outliers or pseudocolor plots.

☒ A numerical value for number of cells or percentage (with statistics) is provided.

Methodology

Sample preparation

PBMCs were selected from stored samples collected from consenting participants recruited into studies coordinated by the laboratory of Professor Alessandro Sette investigating immunodominant peptides associated with responses against pertussis, tuberculosis, dengue, and IgE allergy. The samples were thawed and cultured with 30µg/ml PT (Reagent proteins, USA), 5µg/ml DT (Reagent proteins, USA), 5µg/ml TT (List Biological Laboratories Inc., Campbell, CA), 10 µg/ml phytohaemagglutinin (PHA, Sigma, St Louis, MO, USA), or toxoid diluent (water) at 37oC for 24 hours. The cells were then washed, labelled with an antibody panel for 15 minutes at 4oC before being fixed with paraformaldehyde (Sigma, St Louis, MO, USA).

Instrument

LSRII (Becton, Dickinson and Company, New Jersey, USA)

Software

FlowJo 10.3

Cell population abundance

No sorting was undertaken.

Gating strategy

An exemplar gating is provided in Fig. S4
CD3+/live -> CD3+CD4+ -> non(CD45RA+CCR7+)+ -> CD25+OX40+ and/or CXCR5+

☒ Tick this box to confirm that a figure exemplifying the gating strategy is provided in the Supplementary Information.
